# Natural Nanofibrous Cellulose-Derived Solid Acid Catalysts

**DOI:** 10.34133/2019/6262719

**Published:** 2019-04-16

**Authors:** Zhen-Yu Wu, Peng Yin, Huan-Xin Ju, Zhi-Qin Chen, Chao Li, Si-Cheng Li, Hai-Wei Liang, Jun-Fa Zhu, Shu-Hong Yu

**Affiliations:** ^1^Division of Nanomaterials & Chemistry, Hefei National Laboratory for Physical Sciences at the Microscal, CAS Center for Excellence in Nanoscience, Hefei Science Center of CAS, Collaborative Innovation Center of Suzhou Nano Science and Technology, Department of Chemistry, University of Science and Technology of China, Hefei 230026, China; ^2^National Synchrotron Radiation Laboratory, University of Science and Technology of China, Hefei 230026, China

## Abstract

Solid acid catalysts (SACs) have attracted continuous research interest in past years as they play a pivotal role in establishing environmentally friendly and sustainable catalytic processes for various chemical industries. Development of low-cost and efficient SACs applicable to different catalysis processes are of immense significance but still very challenging so far. Here, we report a new kind of SACs consisting of sulfonated carbon nanofibers that are prepared via incomplete carbonization of low-cost natural nanofibrous cellulose followed by sulphonation with sulfuric acid. The prepared SACs feature nanofibrous network structures, high specific surface area, and abundant sulfonate as well as hydroxyl and carboxyl groups. Remarkably, the nanofibrous SACs exhibit superior performance to the state-of-the-art SACs for a wide range of acid-catalyzed reactions, including dimerization of *α*-methylstyrene, esterification of oleic acid, and pinacol rearrangement. The present approach holds great promise for developing new families of economic but efficient SACs based on natural precursors via scalable and sustainable protocols in the future.

## 1. Introduction

Acid catalysis plays a crucial role in many industrial chemical processes, such as fine-chemical synthesis, oil refining, and biomass conversions [1–7]. Traditional liquid acid catalysts, e.g., H_2_SO_4_, HCl, H_3_PO_4_, and HF, are highly efficient and widely used in these processes [2, 5, 7, 8]. Nevertheless, liquid acid catalysts suffer from problems of safety threat, corrosivity to equipments, catalyst waste, and difficulty of separation and recovery, which significantly limit their practical applications [2–4, 8]. The principles of green and sustainable chemistry stimulate the replacement of the homogeneous liquid acid catalysts with heterogeneous solid acid catalysts (SACs) with inherent advantages of being safe, environmentally friendly with respect to corrosiveness, less waste, and ease of separation and recovery [2, 5, 8–10]. Yet the catalytic performance of the state-of-the-art SACs, such as heteropolyacids, metal oxides (e.g., niobic acid), phosphates, zeolite (e.g., H-Mordenite), and sulfonated resins (e.g., Amberlyst-15, nafion) [1, 8], needs to be significantly improved owing to their low densities of acid sites, poor stability, and high cost [7, 8].

To this end, some novel SACs, such as phenylene-sulfonic acid functionalized ethenylene-silica [11], sulfonated polymers [12], carbon-silica composites [13, 14], and carbon-based materials [15–22], have been developed recently and showed promising performance for various acid-catalyzed reactions. A particularly important contribution to this field is the development of carbonaceous SACs by the sulfonation of incompletely carbonized D-glucose and sucrose by Hara et al. [16]. The incompletely carbonized carbohydrates with flexible polycyclic carbon frameworks and large amount of oxygen-based groups are beneficial to the followed sulfonation process [4, 16, 18, 20, 23]. As a result, the prepared SACs contain abundant -SO_3_H acid sites as well as high density of hydrophilic functional groups and thus show good catalytic performance in some hydrophilic reactions, such as hydration, hydrolysis, and esterification reactions [4, 16, 18, 20, 23]. However, the low specific surface area and poor porosity of carbohydrate-derived carbonaceous SACs hinder the diffusion of hydrophobic reactants to active acid sites and therefore result in poor even no catalytic activity of these catalysts for hydrophobic reactions [13, 24–26]. Developing carbon-based SACs with high -SO_3_H density, porous nanostructure, and large specific surface area is therefore highly desirable but challenging to promise these catalysts applicable to both hydrophilic and hydrophobic reactions as well as other important reactions [24–26].

Herein, we report a new type of highly porous carbon-based SACs that are prepared via a two-step process of carbonization and sulfonation of natural nanofibrous cellulose. Owing to the low-cost natural cellulose precursor and simple preparation process, our method is environmentally friendly, inexpensive, and easy to scale up. Importantly, the prepared SACs well inherit the three-dimensional (3D) nanofiber network structure of natural cellulose precursor and thus exhibit high specific surface areas (up to 837 m^2^ g^−1^) and large pore volumes (up to 0.92 cm^3^ g^−1^); the efficient sulfonation process endows the nanofibrous SACs with abundant Brönsted acid sites including -SO_3_H groups (up to 2.42 mmol g^−1^) as well as hydroxyl (-OH) and carboxyl (-COOH) groups (total acid density of up to 3.88 mmol g^−1^). As a result, in a wide range of important acid-catalyzed reactions, including dimerization of *α*-methylstyrene (AMS, a hydrophobic acid-catalyzed reaction), esterification of oleic acid (a hydrophilic acid-catalyzed reaction), and pinacol rearrangement (an acid strength-dependent reaction), our nanofibrous SACs exhibit much better performance than the state-of-the-art SACs, e.g., Amberlyst-15, H-Mordenite and niobic acid, and in some cases even better than H_2_SO_4_ under similar reaction conditions.

## 2. Results

### 2.1. Preparation and Structural Characterizations of Nanofibrous SACs

**Figure 1:**
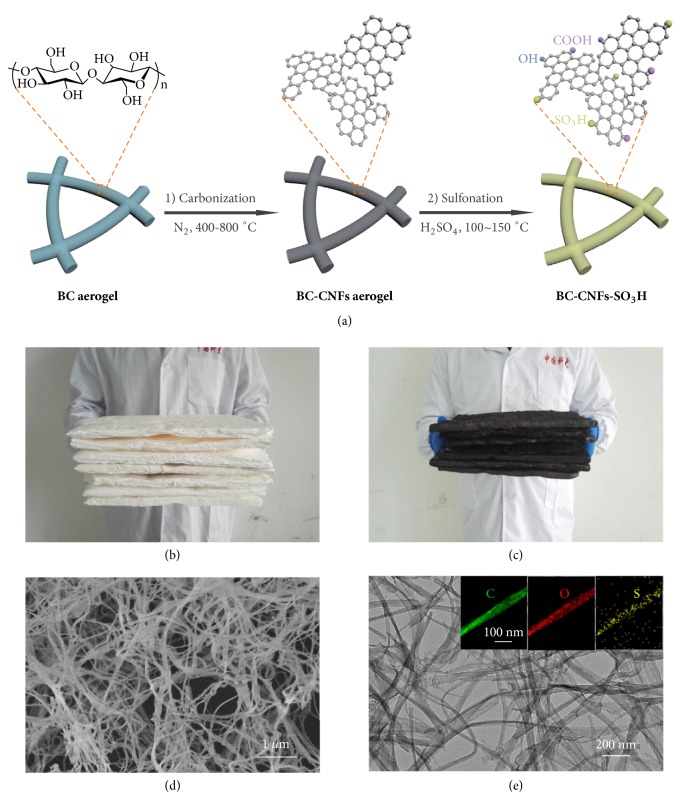
**Preparation and structural characterizations of nanofibrous SACs.** (a) Schematic illustration for the preparation processes of BC-CNFs-SO_3_H SACs. Digital images of large-scale preparation of (b) BC and (c) BC-CNFs aerogels. (d) SEM and (e) TEM images of BC-CNFs-400-SO_3_H. The insets in (e) are elemental mapping images for an individual nanofiber.

We recently demonstrated two kinds of natural nanofibrous celluloses, i.e., bacterial cellulose (BC) and wood-based nanofibrillated cellulose (NFC), as ideal precursors for preparation of carbonaceous nanofiber aerogels [27–29]. BC, a low-cost biomass consisting of 3D nanofibrous cellulose network that can be now produced on an industrial scale via a microbial fermentation process [27], was selected as a typical precursor in this work for preparing nanofibrous SACs. The preparation of nanofibrous SACs involves a two-step process of (1) carbonization of BC aerogels at 400-800°C under a N_2_ atmosphere to generate carbon-based nanofibers (BC-CNFs) aerogels and (2) subsequent sulfonation of the BC-CNFs with concentrated H_2_SO_4_ or fuming H_2_SO_4_ (Figure 1(a)). The obtained catalysts are denoted as BC-CNFs-*x*-SO_3_H or BC-CNFs-*x*-SO_3_H*∗*, where *x* is the pyrolysis temperature and asterisk indicates the SACs prepared with fuming H_2_SO_4_. The commercial availability of low-cost BC precursors and simple preparation processes allow BC-CNFs-SO_3_H catalysts to be easily scaled up (Figures 1(b) and 1(c)). Scanning electron microscopy (SEM) and transmission electron microscopy (TEM) images clearly indicate that the nanofibrous network structures of original BC precursors are kept well in all of the BC-CNFs-*x*-SO_3_H and BC-CNFs-*x*-SO_3_H*∗* catalysts even after carbonization and harsh sulfonation processes (Figures 1(d) and 1(e) and Figures [Supplementary-material supplementary-material-1]-[Supplementary-material supplementary-material-1]). The effective sulfonation is roughly confirmed by energy dispersive X-ray spectroscopy (EDS) spectra ([Supplementary-material supplementary-material-1]). The corresponding elemental mapping images show that S and O species are homogeneously distributed throughout the individual nanofiber as well as the whole nanofibrous network structure (the insets of Figure 1(e) and Figures [Supplementary-material supplementary-material-1]-[Supplementary-material supplementary-material-1]), implying that -SO_3_H groups have been successfully grafted onto BC-CNFs and were uniformly distributed within the whole nanofibrous network. The XRD patterns of BC-CNFs-*x*-SO_3_H show broad but weak diffraction peaks (2*θ* = 10-30°, [Supplementary-material supplementary-material-1]), indicating the amorphous carbon structure composed of aromatic carbon sheets in a considerably random fashion, which is consistent with Raman analysis results ([Supplementary-material supplementary-material-1]) [18].

N_2_ sorption measurements were performed to investigate the textural properties of BC-CNFs-SO_3_H SACs and the results were summarized in Table 1 and Figures [Supplementary-material supplementary-material-1] and [Supplementary-material supplementary-material-1]. All of BC-CNFs-*x*-SO_3_H SACs show high specific surface area ranging from 496 to 837 m^2^ g^−1^ and large pore volume ranging from 0.41 to 0.92 cm^3^ g^−1^. These values are much higher than those of previously reported biomass-derived SACs, such as D-glucose [23], sucrose [16], and microcrystalline cellulose derived SACs [18]. Note that the sulfonation with concentrated H_2_SO_4_ did not damage the porous structure of BC-CNFs, while the fuming H_2_SO_4_ sulfonation led to a distinct decrease of specific surface area; the specific surface area considerably dropped from 512 m^2^ g^−1^ for BC-CNFs-400 to 192 m^2^ g^−1^ for BC-CNFs-400-SO_3_H*∗*. Additionally, the pore size distribution analysis manifests the presence of a large number of mesopores for BC-CNFs-SO_3_H SACs (Figures [Supplementary-material supplementary-material-1] and [Supplementary-material supplementary-material-1]). As far as we know, this work represents the first case of preparing SACs with high specific surface area and large pore volume from biomass precursors without templating or activation processes.

### 2.2. Surface Chemistry Characterization of Nanofibrous SACs

We then carried out the Fourier transform infrared spectroscopy (FT-IR), ^13^C magic angle spinning nuclear magnetic resonance (^13^C MAS NMR), acid-base back titration, X-ray photoelectron spectroscopy (XPS) and X-ray absorption spectroscopy (XAS) analyses to reveal the detailed surface chemistry of the nanofibrous SACs.

**Figure 2:**
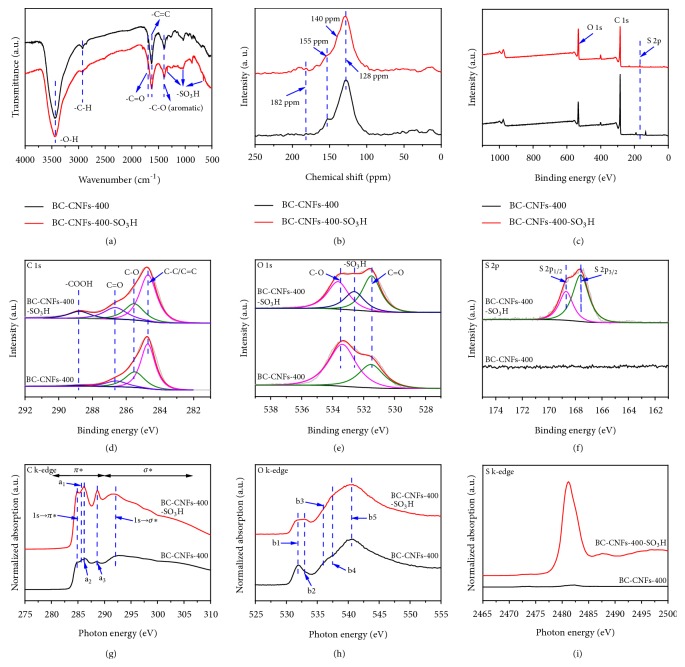
**Surface chemistry characterization of nanofibrous SACs.** (a) FT-IR spectra, (b) ^13^C MAS NMR spectra and (c) XPS survey spectra of BC-CNFs-400 and BC-CNFs-400-SO_3_H. (d) C 1s, (e) O 1s and (f) S 2p high resolution XPS spectra of BC-CNFs-400 and BC-CNFs-400-SO_3_H. (g) C K-edge, (h) O K-edge, and (i) S K-edge XAS spectra of BC-CNFs-400 and BC-CNFs-400-SO_3_H.

Figure 2(a) shows the FT-IR spectra of BC-CNFs-400 before and after sulfonation. For BC-CNFs-400, the peaks at 3000-3700 cm^−1^, 2880-2990 cm^−1^ and 1350-1400 cm^−1^ are assigned to O-H, C-H and aromatic C-O stretching vibrations, respectively [30]; The peaks at 1700 cm^−1^ and 1500-1670 cm^−1^ are attributed to C=O and C=C stretching vibration, respectively [30]. The rich oxygen-containing groups in BC-CNFs-400 are beneficial to followed sulfonation processes. After sulfonation, three new peaks at 1338 cm^−1^, 1065 cm^−1^ and 658 cm^−1^ attributed to -SO_3_H groups emerge in the FT-IR spectrum of BC-CNFs-400-SO_3_H [18, 30]. The surface functional groups on the nanofibers were further analyzed by ^13^C MAS NMR (Figure 2(b) and [Supplementary-material supplementary-material-1]). The peaks at 128, 155, and 182 ppm assignable to polycyclic aromatic carbon atoms, phenolic OH, and COOH groups are found in the both ^13^C MAS NMR spectra of BC-CNFs-*x* and BC-CNFs-*x*-SO_3_H as well as BC-CNFs-400-SO_3_H*∗* [13, 18, 23]. Meanwhile, all of BC-CNFs-SO_3_H SACs exhibit a shoulder peak at 140 ppm, which belongs to the aromatic carbon with SO_3_H groups (Ar-SO_3_H, ca. 140 ppm) [23, 24]. The abundant oxygen-containing groups make these nanofibrous SACs have an excellent hydrophilic property ([Supplementary-material supplementary-material-1]).

Further, the total acid density (referring to total H^+^ number per gram of Brönsted acids, i.e., the total density of SO_3_H, COOH, and OH groups) of the nanofibrous SACs was estimated by acid-base back titration method; the SO_3_H density was determined by elemental analyses based on the fact that sulfur element exclusively came from -SO_3_H group in the nanofibrous SACs. The results of the nanofibrous SACs as well as H_2_SO_4_ and several reference SACs were summarized in Table 1. The BC-CNFs-400-SO_3_H possesses a high total acid density of 1.84 mmol g^−1^ and SO_3_H density of 0.85 mmol g^−1^, comparable to that of Nafion R-1100 (1.13 mmol g^−1^ and 1.02 mmol g^−1^, respectively). With the increase of carbonization temperature, the total acid density and SO_3_H density drop dramatically for BC-CNFs-600-SO_3_H and BC-CNFs-800-SO_3_H because of the increased difficulty in sulfonation for the carbons with higher carbonization degree [24]. Of note, BC-CNFs-400-SO_3_H∗ prepared with fuming H_2_SO_4_ sulfonation exhibits a much higher total acid density of 3.88 mmol g^−1^ and SO_3_H density of 2.42 mmol g^−1^; the SO_3_H density surpasses most of reported carbon-based SACs [13, 16–19, 23–26] and even can compete with Amberlyst-15 with a high SO_3_H density of 4.36 mmol g^−1^.

The surface composition and chemical state of the constituent elements of BC-CNFs-*x* and corresponding SACs were additionally characterized by XPS (Figures 2(c)–2(f) and [Supplementary-material supplementary-material-1]). The C1s spectrum of BC-CNFs-400 can be deconvoluted into four peaks at 284.8 eV, 285.5 eV, 286.7 eV, and 288.8 eV corresponding to C-C and/or C=C, C-O, C=O, and COOH, respectively (Figure 2(d)) [31, 32]. Interestingly, the intensity of C=O and COOH peaks increases obviously in BC-CNFs-400-SO_3_H, probably because of the oxidization of carbon during the sulfonation process. In the high-resolution O1s spectra, the peaks at 531.5 eV and 533.4 eV in BC-CNFs-400 are assigned to oxygen atoms in C=O and C-O, respectively (Figure 2(e)) [30]. A new O1s peak centered at 532.6 eV appears for BC-CNFs-400-SO_3_H, which can be attributed to oxygen atoms in -SO_3_H groups [30]. The high-resolution S2p spectrum of BC-CNFs-400-SO_3_H can be deconvoluted into two peaks at 167.6 eV and 168.7 eV (Figure 2(f)), corresponding to S2p_3/2_ and S2p_1/2_ in -SO_3_H groups, respectively [30]. No other sulfur-containing configurations are observed by XPS analyses. The XPS of other nanofibrous SACs were also analyzed and the results were presented in [Supplementary-material supplementary-material-1].

XAS can probe unoccupied electronic states and is particularly sensitive to the local electronic configuration. XAS measurements of C, O, and S K-edge were therefore performed to further investigate the electronic structure of the nanofibrous SACs. The C K-edge XAS spectra of BC-CNFs-400 and BC-CNFs-400-SO_3_H clearly show the presence of unoccupied *π*∗ (1s→*π*∗) and *σ*∗ (1s→*σ*∗) states at around 284.9 and 292.1 eV, respectively, indicating the aromaticity of both samples (Figure 2(g)) [33]. However, no recognizable structures can be identified between 293 and 310 eV, probably because the amorphous nature of these two samples induces a spread of the *σ*∗ resonances [34]. The peaks at photon energies of 285.7, 286.3, and 288.7 eV are associated with various functional oxygen-containing groups in BC-CNFs-400 and BC-CNFs-400-SO_3_H [33, 34]. The peaks of a1, a2, and a3 correspond to the *π*∗ resonances of carbons being in a chemical bond with oxygen in hydroxyl groups (C-OH), carbons in epoxy groups (C-O-C), and carbons in the double bonds (C=O) in carbonyl and carboxyl (C=O, COOH), respectively [33–35]. Remarkably, the peak of a3 of BC-CNFs-400-SO_3_H is much sharper and stronger than that of BC-CNFs-400, indicating much increased contents of carbonyl and carboxyl groups after sulfonation, agreeing with the XPS results well. The O K-edge XAS spectra further reveal the changes of surface chemistry during sulfonation process. BC-CNFs-400 and BC-CNFs-400-SO_3_H show very similar peaks at 531.8 (b1), 536.0 (b3) and 540.5 eV (b5), which can be assigned to the *π*∗ resonance of C=O/COOH bonds, *σ*∗ resonances of C-OH bonds and C=O/COOH bonds, respectively (Figure 2(h)) [35, 36]. Two new peaks at photon energies of 532.9 and 537.5 eV emerge at O K-edge XAS spectrum of BC-CNFs-400-SO_3_H, which is associated with the *π*∗ and *σ*∗ resonances of -SO_3_H groups [37]. Finally, the S K-edge XAS spectra directly reveal the successful sulfonation: BC-CNFs-400-SO_3_H displays a peak at 2481.2 eV that is assigned to -SO_3_H groups with S in the +6 state [38], while BC-CNFs-400 has no peak at 2470-2495 eV (Figure 2(i)). The C, O, and S K-edge of other nanofibrous SACs were also tested and carefully analyzed; all of them exhibit similar results with BC-CNFs-400-SO_3_H ([Supplementary-material supplementary-material-1]).

Overall, the above FT-IR, ^13^C MAS NMR, acid density test, and XPS and XAS analyses indicate unambiguously that sulfonation can efficiently endow the incompletely carbonized natural nanofibrous cellulose with abundant -SO_3_H groups and other oxygen-containing groups, meanwhile maintaining the nanofibrous structures and high specific surface areas.

### 2.3. The Performance of Acid-Catalyzed Reactions

Three different but highly important acid-catalyzed liquid-phase reactions were selected for evaluating the catalytic performance of our nanofibrous SACs, including a hydrophobic acid-catalyzed reaction (dimerization of AMS), a hydrophilic acid-catalyzed reaction (esterification of oleic acid with methanol), and an acid strength-dependent reaction (pinacol rearrangement). H_2_SO_4_ and several state-of-the-art SACs, i.e., Amberlyst-15, Nafion R-1100, H-ZSM-5, H-mordenite, and niobic acid, were also tested under the same conditions for comparison.

### 2.4. Catalytic Dimerization of AMS

The dimerization of AMS produces valuable unsaturated dimers (2,4-diphenyl-4-methyl-1-pentene and 2,4-diphenyl-4-methyl-2-pentene) accompanied with an undesirable saturated dimer (1,1,3-trimethyl-3-phenylindan) [13, 25]. The unsaturated dimers are industrially important chemicals in the syntheses of styrene-butadiene-rubber and acrylonitrile-butadiene-styrene resin; thus it is necessary to selectively synthesize the unsaturated dimers by inhibiting the formation of the saturated dimer [13, 25]. H_2_SO_4_ gave a nearly 100% conversion but a very poor selectivity; 95.7% AMS was converted into the undesirable saturated dimer (Table 2). The time courses of conversion and yield tests show that most of AMS was rapidly converted into unsaturated and saturated dimers during 0.5 h over H_2_SO_4_. Unfortunately, the unsaturated dimers were gradually converted into saturated dimers at following reaction process; finally the yield of unsaturated dimers was decreased to 4.3% after 20 h ([Supplementary-material supplementary-material-1]), indicating that the intramolecular Friedel-Crafts alkylation proceeded continuously over H_2_SO_4_. As a conventional resin-based strong solid acid, Nafion R-1100 with high density of SO_3_H groups could not efficiently catalyze the AMS dimerization, which is associated with its low specific surface area [13], while Amberlyst-15 with relatively large surface area exhibited a superior catalytic conversion of 98.8% but a low selectivity of 56.3% for unsaturated dimers (Table 2). The whole reaction process of the dimerization of AMS for Amberlyst-15 was very similar to that of H_2_SO_4_ ([Supplementary-material supplementary-material-1]), but its reaction speed was much slow than that of H_2_SO_4_, probably because of its low SO_3_H density. In addition, H-ZSM-5, H-Mordenite and niobic acid with acidic OH groups also displayed negligible catalytic activity for the dimerization of AMS (lower that 10% conversion).

Remarkably, when the BC-CNFs-*x*-SO_3_H catalysts were tested, they showed remarkable catalytic performance for the AMS dimerization. Particularly, the conversion of AMS and selectivity for unsaturated dimers could reach 96.3% and 96.8% for BC-CNFs-400-SO_3_H and 92.9% and >99% for BC-CNFs-600-SO_3_H after 20 h (Table 2). The much higher selectivity of BC-CNFs-*x*-SO_3_H to the unsaturated dimers over H_2_SO_4_ and Amberlyst-15 is probably due to the blocking of intramolecular Friedel-Crafts alkylation on BC-CNFs-*x*-SO_3_H enriched with phenolic OH groups ([Supplementary-material supplementary-material-1]) [25]. Indeed, the incorporation of* p*-cresol in the H_2_SO_4_-catalyzed AMS dimerization could distinctly improve the selectivity to unsaturated dimers ([Supplementary-material supplementary-material-1]). Of note, when using microcrystalline cellulose and glucose derived SACs (i.e., cellulose-600-SO_3_H and glucose-600-SO_3_H) with low specific surface area of <1 m^2^ g^−1^ for catalyzing the dimerization of AMS, very low activity (lower than 5% conversion) was observed despite their high total acid density and SO_3_H density (Figures [Supplementary-material supplementary-material-1] and [Supplementary-material supplementary-material-1]). These results clearly manifest the vital role of the high porosity resulting from the nanofibrous structure of BC-CNFs-*x*-SO_3_H in hydrophobic acid-catalyzed reaction.

The reusability of BC-CNFs-600-SO_3_H for AMS dimerization was evaluated by a simple decantation method ([Supplementary-material supplementary-material-1]). No obvious decrease in activity and selectivity was observed after three reuses of the same sample, though the activity decreased in the fourth cycle. We found that the nanofibrous microstructure of BC-CNFs-600-SO_3_H was well retained ([Supplementary-material supplementary-material-1]), but the total acid density dropped to 0.51 mmol g^−1^, which should be an important reason for the catalytic performance degradation. Besides, the blocking of active SO_3_H groups by adsorbed reactants and/or products and the unavoidable catalyst loss during the recycle process should be considered as other reasons for the decrease of catalytic performance [25, 39].

### 2.5. Catalytic Esterification of Oleic Acid with Methanol

The esterification of fatty acids with methanol was selected to further demonstrate the wide applicability of BC-CNFs-*x*-SO_3_H for various acid-catalyzed reactions (Table 3). The esterification of fatty acids with alcohols to produce fatty acid esters is an important step in the production of biodiesel, which is considered a promising alternative fuel to replace petroleum-based fuels because of its biodegradability, nontoxicity, and favorable combustion emission profile [4, 16, 22]. Methyl oleate was only product detected by gas chromatography in this acid-catalyzed liquid-phase reaction. When three conventional SACs including H-ZSM-5, H-Mordenite, and niobic acid which are free of -SO_3_H were used for esterification of oleic acid with methanol, the conversion of oleic acid was very low (<15%) after 6 h reaction and the corresponding turnover frequencies (TOFs) were less than 1 h^−1^. Under the same conditions, Nafion R-1100 with high SO_3_H density also showed a poor activity, while Amberlyst-15 with higher SO_3_H density and larger specific surface area gave a moderate activity with a conversion of 42.4% and a TOF of 8.86 h^−1^. Remarkably, BC-CNFs-400-SO_3_H and BC-CNFs-600-SO_3_H with relatively low SO_3_H density even exhibited much better catalytic performance; ca. 90% oleic acid was converted on them within 12 h and their TOFs are more than 15.0 h^−1^. The higher catalytic activity of BC-CNFs-SO_3_H over Nafion R-1100 and Amberlyst-15 is attributed to not only their high specific surface areas but also the flexibility carbon structures with large amounts of hydrophilic functional groups that provide access for the reactant molecules to the active SO_3_H groups inside the carbon matrix [18, 23]. As expected, H_2_SO_4_, as a homogeneous acid catalyst, had the highest activity with a conversion of 98.3% and a TOF of 41.6 h^−1^. When BC-CNFs-400-SO_3_H∗ with higher SO_3_H density (nearly triple of that in BC-CNFs-400-SO_3_H) which was prepared by fuming H_2_SO_4_ sulfonation was used for this esterification reaction, it showed nearly the same conversion and comparable TOF (29.3 h^−1^) as the high-performance H_2_SO_4_ catalyst. Furthermore, BC-CNFs-400-SO_3_H could be well recycled for four times without obvious performance degradation ([Supplementary-material supplementary-material-1]).

### 2.6. Catalytic Pinacol Rearrangement

Pinacol rearrangement is a valuable process for synthesizing aldehydes or ketones through the skeletal rearrangement of 1,2-diols and elimination of water [40]. The reaction involves the evolution of a cationic intermediate, which is stabilized under highly acidic conditions [11]. We therefore selected this reaction to show the association of acid strength of the BC-CNFs-SO_3_H catalysts with their catalytic performance (Table 4). H-ZSM-5, H-Mordenite, niobic acid, and Nafion R-1100 showed very poor conversion (lower that 10%), as results of their low acid strength. In contrast, all of BC-CNFs-SO_3_H catalysts exhibited high performance for pinacol rearrangement with >85% conversion and >70% selectivity for pinacolone. The BC-CNFs-400-SO_3_H catalyst could be recycled for three times with a slight performance degradation ([Supplementary-material supplementary-material-1]). Particularly, BC-CNFs-400-SO_3_H∗ showed a conversion of 99.4% and a selectivity for pinacolone of 81.9%, which is very close to H_2_SO_4_ and exceeds Amberlyst-15. The high acid strength of BC-CNFs-400-SO_3_H∗ was verified by ^31^P MAS NMR tests ([Supplementary-material supplementary-material-1]).

### 2.7. Catalytic Performance for Other Reactions

Besides being used for the above acid-catalyzed reactions, the BC-CNFs-SO_3_H catalysts were also explored for other liquid-phase reactions, including the synthesis of *β*-enamino ketones/esters and hydrogenation of nitrobenzene. Enamination of *β*-dicarbonyl compounds, an important and widely used transformation, can form *β*-enamino ketones and esters [41, 42]. The obtained compounds are a highly versatile class of intermediates for the synthesis of heterocycles and biologically active compounds [41]. Various amines (aromatic, cyclic, and aliphatic) and *β*-diketones or *β*-ketoesters were investigated for synthesizing *β*-enamino ketones/esters in the presence of BC-CNFs-400-SO_3_H ([Supplementary-material supplementary-material-1]). Most yields of *β*-enamino ketones/esters exceeded 90%, which was close comparable with the results of reported metal-based catalysts [41]. The relatively low yield (52.5%) of the reaction between acetylacetone and* o*-chloroaniline probably results from the stereo-hindrance effect. The high catalytic performance of BC-CNFs-400-SO_3_H for synthesis of *β*-enamino ketones/esters is believed to be associated with high solid acid groups and active defect sites [42]. In addition, BC-CNFs-400-SO_3_H also exhibited promising activity for catalyzing the hydrogenation of nitrobenzene. Using N_2_H_4_·H_2_O as the reductant, nitrobenzene could completely converted into aniline over BC-CNFs-400-SO_3_H with a superior yield of 99.6%, which suppresses most of the reported carbon-based catalysts ([Supplementary-material supplementary-material-1]). The BC-CNFs-400-SO_3_H catalyst could be well recycled; only a slight activity decrease (ca. 8%) was observed after 5 runs of catalytic reactions ([Supplementary-material supplementary-material-1]), demonstrating good reusability as a heterogeneous carbon-based catalyst.

These catalysis tests clearly confirm the distinct advantages of BC-CNFs-SO_3_H SACs over H_2_SO_4_ and the state-of-the-art SACs in a wide range of liquid-phase acid-catalyzed reactions. The high performance of BC-CNFs-SO_3_H SACs is associated with their unique nanofibrous network structure, abundant functional groups, high -SO_3_H density, and acid strength. Another superiority of BC-CNFs-SO_3_H catalysts is easy to scale up due to the commercially available low-cost biomass precursors and simple preparation processes. Particularly, the more sustainable and cheaper wood-based NFC could also be employed as precursor to prepare efficient nanofibrous SACs ([Supplementary-material supplementary-material-1]). Moreover, our developed method can also be extended to other nanofibrous SACs, such as phosphorylated BC-CNFs ([Supplementary-material supplementary-material-1]).

## 3. Discussion

In summary, we report a facile, low-cost, and scalable method to prepare a new type of nanofibrous SACs by incomplete carbonization and sulfonation of natural nanofibrous celluloses. The prepared nanofibrous SACs showed distinctly better catalytic performance than the state-of-the-art SACs in various important acid-catalyzed liquid-phase reactions, including both hydrophilic and hydrophobic acid-catalyzed reactions. The superior performance of the nanofibrous SACs is associated to their unique structural feature and surface chemistry, i.e., high specific surface areas, large pore volumes, and high SO_3_H density and total acid density. The concept of converting nanofibrous cellulose to SACs demonstrated in this work sheds new light on the further development of highly efficient catalysts based on nanostructured biomass for green and sustainable chemistry.

## 4. Materials and Methods

### 4.1. Materials and Chemicals

Raw materials of purified BC pellicles with fiber content of ~1% (vol/vol) were kindly provided by Ms C. Y. Zhong (Hainan Yeguo Foods Co. Ltd, Hainan, China). The fuming sulfuric acid (50 wt% SO_3_) was purchased from Nanjing Chemical Reagent Co. Ltd (Nanjing, China). Amberlyst-15, Nafion R-1100, and niobic acid were obtained from Puzhen Biological Technology Co. Ltd., Alfa Aesar (China) Chemicals Co. Ltd., and Macklin Biochemical Co. Ltd. (Shanghai, China), respectively. H-ZSM-5 and H-Mordenite were purchased from Shengtan Environmental New Materials Co. Ltd (Shanghai, China). Other chemicals were of analytical grade, commercially available from Sinopharm Chemical Reagent Co. Ltd (Shanghai, China), and used as received without further purification.

### 4.2. Preparation of BC-CNFs-S*O*_3_H SACs

The as-received purified BC pellicles with a thickness of 1 cm were first soaked with deionized water for 3 days and cut into small pieces. Then, the small BC pieces were immersed in 10 mM NH_4_H_2_PO_4_ solution for 3 days and freeze-dried to obtain BC aerogels. The BC aerogels were subsequently heated to 400-800°C under a N_2_ atmosphere with at a heating rate of 5°C min^−1^ speed and kept for 2 h to generate BC-CNFs aerogels. The BC-CNFs aerogels were then sulfonated using concentrated H_2_SO_4_ at 150°C or fuming H_2_SO_4_ (50 wt% SO_3_) at 100°C for 12 h, respectively, before cooling to room temperature and washing with hot distilled water (>80°C) until impurities, such as sulfate ions, were no longer detected in the wash water. Finally, the sulfonated products were freeze-dried to obtain BC-CNFs-SO_3_H SACs. The cellulose-600-SO_3_H and glucose-600-SO_3_H as reference samples were prepared by the same processes using concentrated H_2_SO_4_ for sulfonation. Of note, the precursor of glucose-600-SO_3_H, i.e., glucose, has not been impregnated with deionized water and 10 mM NH_4_H_2_PO_4_ solution before use, due to the high solubility in water.

### 4.3. Characterization

SEM images were taken with a Zeiss Supra 40 scanning electron microscope at an acceleration voltage of 5 kV. TEM images were obtained using a Hitachi H7650 or Hitachi H7700 transmission electron microscope with a CCD imaging system and an accelerating voltage of 100 kV. XRD data was collected on a Philips X'Pert PRO SUPER X-ray diffractometer equipped with graphite monochromatic Cu K*α* radiation (*λ* = 1.54056 Å). EDS spectrum and corresponding elemental mapping were acquired using a Talos F200X transmission electron microscope at an accelerating voltage of 200 kV equipped with an energy dispersive detector. FT-IR spectroscopy was measured on a Bruker Vector 22 Fourier transform infrared spectrometer. ^13^C MAS NMR spectra were recorded on a Bruker AVANCE 400WB NMR spectrometer (400 MHz). N_2_ sorption analysis was carried out using an ASAP 2020 accelerated surface area and porosimetry instrument (Micromeritics), equipped with automated surface area, at 77 K using Barrett-Emmett-Teller (BET) calculations for the surface area. The pore size distribution plot was analyzed from the adsorption branch of the isotherm based on the Barrett-Joyner-Halenda (BJH). Raman scattering spectra were recorded with a Renishaw System 2000 spectrometer using the 514.5 nm line of Ar+ for excitation. XPS was performed on an X-ray photoelectron spectrometer (ESCALab MKII) with an excitation source of Mg K*α* radiation (1253.6 eV). The XAS tests of C K-edge and O K-edge were carried out at the BL11U beamline of National Synchrotron Radiation Laboratory (NSRL, Hefei, China). The S K-edge was performed at the beamline 4B7A beamline of Beijing Synchrotron Radiaition Facility (BSRF, Beijing, China). The XAS spectra in the figures have been normalized to the background before and after the main features. The GC instrument was equipped with a Restek-5 capillary column (5% diphenyl and 95% dimethylsiloxane, 0.32 mm diameter, 60 m length) and a flame ionization detector (FID). Gas chromatograph (GC, Shimadzu GC-2014) was used to calculate the conversion and selectivity of the catalytic products.

### 4.4. Catalytic Reactions

Dimerization of AMS was performed in a 5 mL glass reactor sealed with a Teflon lid. AMS (1 ml) was reacted over the catalyst (5 mg) at 100°C for 20 h unless otherwise specified. After cooling to room temperature, *n*-heptadecane was added as an internal standard and then ethanol (10 ml) as a diluent was added. Samples were withdrawn at intervals from the reaction mixtures and analyzed by gas chromatography using a capillary column.

Esterification of higher fatty acids was performed in a mixture containing 0.010 mol methanol, 0.001 mol oleic acid, 1 ml* n*-octane mixture, and 5 mg catalyst at 80°C for 6 h. After cooling to room temperature,* n*-heptadecane was added as an internal standard and then* n*-octane (10 ml) as a diluent was added. The liquid phase was separated from the reaction mixtures and analyzed by gas chromatography using a capillary column.

Pinacol-pinacolone rearrangement was carried out in the liquid phase as follows. 5 mg catalyst, pinacol (1.0575 mol), and 1 ml water were added in glass reactor. Then they were heated to 130°C and kept for 12 h. After cooling to room temperature,* n*-heptadecane was added as an internal standard and toluene (10 ml) as a diluent was also added. At last, the liquid phase was analyzed using gas chromatography with capillary columns.

Catalytic synthesis of *β*-enamino ketones/esters was carried out in a round bottom flask. Typically, 10 mg catalyst was added into a reactant mixture of diketone (5 mmol) and amine (5.2 mmol). The reaction was performed in air atmosphere with magnetically stirring at 60°C for 0.1-3 h. The reactants and products were analyzed by gas chromatography, and the yield of all reactions refers to the GC yield.

Hydrogenation of nitrobenzene was performed in the presence of 0.3 g nitrobenzene, 0.85 mL hydrazine monohydrate, and 5 mg catalyst at 100°C for 4 h. After cooling to room temperature, 10 ml methanol (diluent) and* n*-dodecane (internal standard) were added, and the liquid phase was analyzed using gas chromatography with capillary columns.

## Figures and Tables

**Table 1:** The structure and surface properties of BC-CNFs-SO_3_H SACs and reference catalysts.

Catalyst	Specific surface area (m^2^ g^−1^)	Pore volume (cm^3^ g^−1^)	Total acid density (mmol g^−1^)^a^	SO_3_H density (mmol g^−1^)^b^

BC-CNFs-400-SO_3_H	496	0.41	1.84	0.85
BC-CNFs-600-SO_3_H	686	0.76	1.26	0.43
BC-CNFs-800-SO_3_H	837	0.92	1.08	0.32
BC-CNFs-400-SO_3_H∗	192	0.42	3.88	2.42
BC-CNFs-400	512	0.38	0.58	/
H_2_SO_4_	/	/	20.4	/
Amberlyst-15	50	0.2-0.4	4.50	4.36
Nafion R-1100	<1	/	1.13	1.02
H-ZSM-5	462	~0.21	0.62	/
H-Mordenite	387	~0.20	0.44	/
Niobic acid	124	0.02	0.41	/

^a^Total acid density is determined by acid-base back titration;  ^b^SO_3_H densities are estimated from the S content in sample compositions determined by elemental analysis.

**Table 2:** Catalytic performance of the catalysts tested for dimerization of *α*-methylstyrene.

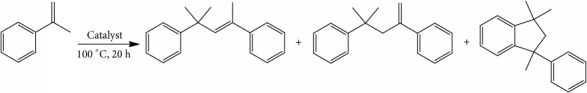				

Entry	Catalyst	Conversion (%)	Selectivity (%)	
			Unsaturated dimers^a^	Saturated dimers^b^

1	BC-CNFs-400-SO_3_H	96.3	96.8	3.20
2	BC-CNFs-600-SO_3_H	92.9	>99%	Trace
3	BC-CNFs-800-SO_3_H	20.7	>95%	Trace
4	BC-CNFs-400-SO_3_H∗	97.7	83.6%	16.4%
5	BC-CNFs-600	Trace	Trace	Trace
6	H_2_SO_4_	ca. 100	4.30	95.7
7	Amberlyst-15	98.8	56.3	43.7
8	Nafion R-1100	Trace	Trace	Trace
9	H-ZSM-5	8.92	>99%	Trace
10	H-Mordenite	6.35	>99%	Trace
11	Niobic acid	Trace	Trace	Trace

^a^2,4-diphenyl-4methyl-1-pentene and 2,4-diphenyl-4-methyl-2-pentene; ^b^1,1,3-trimethyl-3-phenylindan.

**Table 3:** Catalytic performance of the catalysts tested for esterification of oleic acid with methanol.

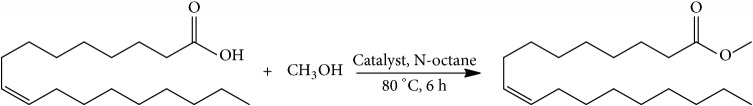			

Entry	Catalyst	Conversion (%)	TOF (h^−1^)^a^

1	BC-CNFs-400-SO_3_H	90.4	18.3
2	BC-CNFs-600-SO_3_H	87.9	15.6
3	BC-CNFs-800-SO_3_H	80.3	9.5
4	BC-CNFs-400-SO_3_H∗	97.2	29.3
5	BC-CNFs-400	19.1	1.23
6	H_2_SO_4_	98.3	41.6
7	Amberlyst-15	42.4	8.86
8	Nafion R-1100	13.2	0.48
9	H-ZSM-5	10.9	0.88
10	H-Mordenite	12.2	0.53
11	Niobic acid	13.3	0.21

^a^The TOF is calculated by the conversion of oleic acid per acid site per hour with conversion less than 40%; the active sites are defined as H^+^ measured by acid-base titration method.

**Table 4:** Catalytic performance of the catalysts tested for pinacol rearrangement.

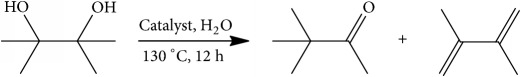				

Entry	Catalyst	Conversion (%)	Selectivity (%)	
			Pinacolone	2,3-Dimethyl-1,3-butadiene

1	BC-CNFs-400-SO_3_H	96.3	78.9	21.1
2	BC-CNFs-600-SO_3_H	92.9	76.7	23.3
3	BC-CNFs-800-SO_3_H	85.6	72.2	27.8
4	BC-CNFs-400-SO_3_H∗	99.4	81.9	18.1
5	BC-CNFs-400	10.2	97.2	2.80
6	H_2_SO_4_	ca. 100	86.5	13.5
7	Amberlyst-15	97.3	65.3	34.7
8	Nafion R-1100	3.20	37.5	62.5
9	H-ZSM-5	8.92	94.6	5.40
10	H-Mordenite	6.35	63.1	36.9
11	Niobic acid	Trace	Trace	Trace

## Data Availability

All data needed to evaluate the conclusions in the paper are present in the paper and the Supplementary Materials. Additional data related to this paper may be requested from the authors.

## Supplementary Materials

Supplementary Materials Figure S1: SEM and TEM images of BC-CNFs-400. Figure S2: SEM and TEM images of BC-CNFs-600 and BC-CNFs-600-SO_3_H. Figure S3: SEM and TEM images of BC-CNFs-800 and BC-CNFs-800-SO_3_H. Figure S4: SEM and TEM images of BC-CNFs-400-SO_3_H∗. Figure S5: EDS spectra of BC-CNFs-400-SO_3_H and BC-CNFs-400-SO_3_H∗. Figure S6: Elemental mapping images of BC-CNFs-400-SO_3_H network. Figure S7: Elemental mapping images for an individual nanofiber of BC-CNFs-400-SO_3_H∗. Figure S8: Elemental mapping of BC-CNFs-400-SO_3_H∗ network. Figure S9: XRD patterns. Figure S10: Raman spectra. Figure S11: N_2_ adsorption-desorption isotherms and pore size distribution curves of BC-CNFs-400 and BC-CNFs-400-SO_3_H. Figure S12: N_2_ adsorption-desorption isotherms and pore size distribution curves of BC-CNFs-600-SO_3_H, BC-CNFs-800-SO_3_H, and BC-CNFs-400-SO_3_H∗. Figure S13: ^13^C MAS NMR spectra. Figure S14: The measurement of contact angles with water for BC-CNFs-400-SO_3_H, BC-CNFs-600-SO_3_H, BC-CNFs-800-SO_3_H, and BC-CNFs-400-SO_3_H∗. Figure S15: C 1s, O 1s, and S 2p high resolution XPS spectra of BC-CNFs-600, BC-CNFs-600-SO_3_H, BC-CNFs-800, BC-CNFs-800-SO_3_H, BC-CNFs-400, and BC-CNFs-400-SO_3_H∗. Figure S16: C K-edge, O K-edge, and S K-edge XAS spectra. Figure S17: Time courses for AMS conversion and yields of unsaturated dimers and saturated dimers. Figure S18: The dimerization of AMS over H_2_SO_4_ in the absence and presence of* p*-cresol (2.4 mL). Figure S19: AMS dimerization performance comparison among glucose-600-SO_3_H, cellulose-600-SO_3_H, and BC-CNFs-600-SO_3_H. Figure S20: Recyclability performance of BC-CNFs-600-SO_3_H for AMS dimerization. Figure S21: TEM images of recycled BC-CNFs-600-SO_3_H after catalyzing AMS dimerization for four times. Figure S22: Recyclability performance of BC-CNFs-400-SO_3_H for esterification of oleic acid with methanol. Figure S23: Recyclability performance of BC-CNFs-400-SO_3_H for pinacol rearrangement. Figure S24: ^31^P MAS NMR spectra of BC-CNFs-400-SO_3_H∗ and some reference SACs. Figure S25: Recyclability performance of BC-CNFs-400-SO_3_H for hydrogenation of nitrobenzene. Figure S26: Characterizations for Wood-CNFs-400-SO_3_H. Figure S27: Characterizations for phosphorylated BC-CNFs-400. Table S1: Catalytic performance of the catalysts tested for synthesis of *β*-Ketoenamines. Table S2: The comparison of catalytic performance of BC-CNF-400-SO_3_H and previously reported carbon-based catalysts for hydrogenation of nitrobenzene. References [9, 18, 43, 44].

## References

[1] Corma A. (1995). Inorganic solid acids and their use in acid-catalyzed hydrocarbon reactions. *Chemical Reviews*.

[2] Corma A. (1997). Solid acid catalysts. *Current Opinion in Solid State & Materials Science*.

[3] Okuhara T. (2002). Water-tolerant solid acid catalysts. *Chemical Reviews*.

[4] Hara M. (2010). Biomass conversion by a solid acid catalyst. *Energy & Environmental Science*.

[5] Takagaki A., Tagusagawa C., Hayashi S., Hara M., Domen K. (2010). Nanosheets as highly active solid acid catalysts for green chemical syntheses. *Energy & Environmental Science*.

[6] Shimizu K.-I., Satsuma A. (2011). Toward a rational control of solid acid catalysis for green synthesis and biomass conversion. *Energy & Environmental Science*.

[7] Gupta P., Paul S. (2014). Solid acids: green alternatives for acid catalysis. *Catalysis Today*.

[8] Liu F., Huang K., Zheng A., Xiao F.-S., Dai S. (2018). Hydrophobic solid acids and their catalytic applications in green and sustainable chemistry. *ACS Catalysis*.

[9] Zhang X., Zhao Y., Xu S. (2014). Polystyrene sulphonic acid resins with enhanced acid strength via macromolecular self-assembly within confined nanospace. *Nature Communications*.

[10] Kitano M., Nakajima K., Kondo J. N., Hayashi S., Hara M. (2010). Protonated titanate nanotubes as solid acid catalyst. *Journal of the American Chemical Society*.

[11] Nakajima K., Tomita I., Hara M., Hayashi S., Domen K., Kondo J. N. (2005). A stable and highly active hybrid mesoporous solid acid catalyst. *Advanced Materials*.

[12] Xing R., Liu N., Liu Y. (2007). Novel solid acid catalysts: Sulfonic acid group-functionalized mesostructured polymers. *Advanced Functional Materials*.

[13] Nakajima K., Okamura M., Kondo J. N. (2009). Amorphous carbon bearing sulfonic acid groups in mesoporous silica as a selective catalyst. *Chemistry of Materials*.

[14] Yue Q., Wang M., Wei J. (2012). A template carbonization strategy to synthesize ordered mesoporous silica microspheres with trapped sulfonated carbon nanoparticles for efficient catalysis. *Angewandte Chemie International Edition*.

[15] Hara M., Yoshida T., Takagaki A. (2004). A carbon material as a strong protonic acid. *Angewandte Chemie International Edition*.

[16] Toda M., Takagaki A., Okamura M. (2005). Green chemistry: biodiesel made with sugar catalyst. *Nature*.

[17] Wang X., Liu R., Waje M. M. (2007). Sulfonated ordered mesoporous carbon as a stable and highly active protonic acid catalyst. *Chemistry of Materials*.

[18] Suganuma S., Nakajima K., Kitano M. (2008). Hydrolysis of cellulose by amorphous carbon bearing SO_3_H, COOH, and OH groups. *Journal of the American Chemical Society*.

[19] Ji J., Zhang G., Chen H. (2011). Sulfonated graphene as water-tolerant solid acid catalyst. *Chemical Science*.

[20] Nakajima K., Hara M. (2012). Amorphous carbon with SO_3_H groups as a solid brønsted acid catalyst. *ACS Catalysis*.

[21] Peng F., Zhang L., Wang H., Lv P., Yu H. (2005). Sulfonated carbon nanotubes as a strong protonic acid catalyst. *Carbon*.

[22] Poonjarernsilp C., Sano N., Tomon H. (2014). Hydrothermally sulfonated single-walled carbon nanohorns for use as solid catalysts in biodiesel production by esterification of palmitic acid. *Applied Catalysis B: Environmental*.

[23] Okamura M., Takagaki A., Toda M. (2006). Acid-catalyzed reactions on flexible polycyclic aromatic carbon in amorphous carbon. *Chemistry of Materials*.

[24] Kitano M., Arai K., Kodama A. (2009). Preparation of a sulfonated porous carbon catalyst with high specific surface area. *Catalysis Letters*.

[25] Suganuma S., Nakajima K., Kitano M. (2011). SO_3_H-bearing mesoporous carbon with highly selective catalysis. *Microporous and Mesoporous Materials*.

[26] Fukuhara K., Nakajima K., Kitano M., Hayashi S., Hara M. (2013). Synthesis and acid catalysis of zeolite-templated microporous carbons with SO_3_H groups. *Physical Chemistry Chemical Physics*.

[27] Wu Z.-Y., Liang H.-W., Chen L.-F., Hu B.-C., Yu S.-H. (2016). Bacterial cellulose: a robust platform for design of three dimensional carbon-based functional nanomaterials. *Accounts of Chemical Research*.

[28] Wu Z.-Y., Li C., Liang H.-W., Chen J.-F., Yu S.-H. (2013). Ultralight, flexible, and fire-resistant carbon nanofiber aerogels from bacterial cellulose. *Angewandte Chemie International Edition*.

[29] Li S., Hu B., Ding Y. (2018). Wood-derived ultrathin carbon nanofiber aerogels. *Angewandte Chemie International Edition*.

[30] Zhao K., Liu S., Li K. (2017). Fabrication of −SO_3_H functionalized aromatic carbon microspheres directly from waste Camellia oleifera shells and their application on heterogeneous acid catalysis. *Molecular Catalysis*.

[31] Gong Y., Wang H., Wei Z., Xie L., Wang Y. (2014). An efficient way to introduce hierarchical structure into biomass-based hydrothermal carbonaceous materials. *ACS Sustainable Chemistry & Engineering*.

[32] Zhang M., Wu M., Liu Q., Wang X., Lv T., Jia L. (2017). Graphene oxide mediated cellulose-derived carbon as a highly selective catalyst for the hydrolysis of cellulose to glucose. *Applied Catalysis A: General*.

[33] Ganguly A., Sharma S., Papakonstantinou P., Hamilton J. (2011). Probing the thermal deoxygenation of graphene oxide using high-resolution in situ X-ray-based spectroscopies. *The Journal of Physical Chemistry C*.

[34] Lenardi C., Piseri P., Briois V., Bottani C. E., Li Bassi A., Milani P. (1999). Near-edge X-ray absorption fine structure and Raman characterization of amorphous and nanostructured carbon films. *Journal of Applied Physics*.

[35] Kuznetsova A., Popova I., Yates J. T. (2001). Oxygen-containing functional groups on single-wall carbon nanotubes: NEXAFS and vibrational spectroscopic studies. *Journal of the American Chemical Society*.

[36] Wolcott A., Schiros T., Trusheim M. E. (2014). Surface structure of aerobically oxidized diamond nanocrystals. *The Journal of Physical Chemistry C*.

[37] Todd E. C., Sherman D. M., Purton J. A. (2003). Surface oxidation of chalcopyrite (CuFeS_2_) under ambient atmospheric and aqueous (pH 2-10) conditions: Cu, Fe L- and O K-edge X-ray spectroscopy. *Geochimica et Cosmochimica Acta*.

[38] Sarret G., Connan J., Kasrai M. (1999). Chemical forms of sulfur in geological and archeological asphaltenes from Middle East, France, and Spain determined by sulfur K- and L-edge X-ray absorption near-edge structure spectroscopy. *Geochimica et Cosmochimica Acta*.

[39] López D. E., Goodwin J. G., Bruce D. A., Lotero E. (2005). Transesterification of triacetin with methanol on solid acid and base catalysts. *Applied Catalysis A: General*.

[40] Chen S.-Y., Lee J.-F., Cheng S. (2010). Pinacol-type rearrangement catalyzed by Zr-incorporated SBA-15. *Journal of Catalysis*.

[41] Sun J., Dong Z., Li P. (2013). Ag nanoparticles in hollow magnetic mesoporous spheres: A highly efficient and magnetically separable catalyst for synthesis of *β*-enaminones. *Materials Chemistry and Physics*.

[42] Deng D., Xiao L., Chung I.-M., Kim I. S., Gopiraman M. (2017). Industrial-quality graphene oxide switched highly efficient metaland solvent-free synthesis of *β*-ketoenamines under feasible conditions. *ACS Sustainable Chemistry & Engineering*.

[43] Fukuhara K., Nakajima K., Kitano M., Kato H., Hayashi S., Hara M. (2011). Structure and catalysis of cellulose-derived amorphous carbon bearing SO_3_H groups. *ChemSusChem*.

[44] Suganuma S., Nakajima K., Kitano M., Hayashi S., Hara M. (2012). Sp^3^-Linked amorphous carbon with sulfonic acid groups as a heterogeneous acid catalyst. *ChemSusChem*.

