# Supplementary material for: Natural Nanofibrous Cellulose-Derived Solid Acid Catalysts
Source: Research (Wash D C). 2019 Apr 16;2019:6262719. doi: 10.34133/2019/6262719 (PMC6750093; doi:10.34133/2019/6262719)
Supplement: Supplementary Materials — Figure S1: SEM and TEM images of BC-CNFs-400. Figure S2: SEM and TEM images of BC-CNFs-600 and BC-CNFs-600-SO3H. Figure S3: SEM and TEM images of BC-CNFs-800 and BC-CNFs-800-SO3H. Figure S4: SEM and TEM images of BC-CNFs-400-SO3H∗. Figure S5: EDS spectra of BC-CNFs-400-SO3H and BC-CNFs-400-SO3H∗. Figure S6: Elemental mapping images of BC-CNFs-400-SO3H network. Figure S7: Elemental mapping images for an individual nanofiber of BC-CNFs-400-SO3H∗. Figure S8: Elemental mapping of BC-CNFs-400-SO3H∗ network. Figure S9: XRD patterns. Figure S10: Raman spectra. Figure S11: N2 adsorption-desorption isotherms and pore size distribution curves of BC-CNFs-400 and BC-CNFs-400-SO3H. Figure S12: N2 adsorption-desorption isotherms and pore size distribution curves of BC-CNFs-600-SO3H, BC-CNFs-800-SO3H, and BC-CNFs-400-SO3H∗. Figure S13: 13C MAS NMR spectra. Figure S14: The measurement of contact angles with water for BC-CNFs-400-SO3H, BC-CNFs-600-SO3H, BC-CNFs-800-SO3H, and BC-CNFs-400-SO3H∗. Figure S15: C 1s, O 1s, and S 2p high resolution XPS spectra of BC-CNFs-600, BC-CNFs-600-SO3H, BC-CNFs-800, BC-CNFs-800-SO3H, BC-CNFs-400, and BC-CNFs-400-SO3H∗. Figure S16: C K-edge, O K-edge, and S K-edge XAS spectra. Figure S17: Time courses for AMS conversion and yields of unsaturated dimers and saturated dimers. Figure S18: The dimerization of AMS over H2SO4 in the absence and presence of p-cresol (2.4 mL). Figure S19: AMS dimerization performance comparison among glucose-600-SO3H, cellulose-600-SO3H, and BC-CNFs-600-SO3H. Figure S20: Recyclability performance of BC-CNFs-600-SO3H for AMS dimerization. Figure S21: TEM images of recycled BC-CNFs-600-SO3H after catalyzing AMS dimerization for four times. Figure S22: Recyclability performance of BC-CNFs-400-SO3H for esterification of oleic acid with methanol. Figure S23: Recyclability performance of BC-CNFs-400-SO3H for pinacol rearrangement. Figure S24: 31P MAS NMR spectra of BC-CNFs-400-SO3H∗ and some reference SACs. Figure S25: Recyclabili [file 6262719.f1.doc]

**Supplementary Materials**

**Natural nanofibrous cellulose-derived solid acid catalysts**

Zhen-Yu Wu,^1,†^ Peng Yin,^1,†^ Huan-Xin Ju,^2^ Zhi-Qin Chen,^1^ Chao Li,^1^ Si-Cheng Li,^1^ Hai-Wei Liang,^1^,* Jun-Fa Zhu,^2^ and Shu-Hong Yu^1^,*

^1^Division of Nanomaterials & Chemistry, Hefei National Research Center for Physical Sciences at the Microscale, CAS Center for Excellence in Nanoscience, Hefei Science Center of CAS, Collaborative Innovation Center of Suzhou Nano Science and Technology, Department of Chemistry, University of Science and Technology of China, Hefei 230026, China

^2^National Synchrotron Radiation Laboratory, University of Science and Technology of China, Hefei 230026, China

^†^These authors contributed equally to this work.

*Corresponding author: hwliang@ustc.edu.cn; shyu@ustc.edu.cn.


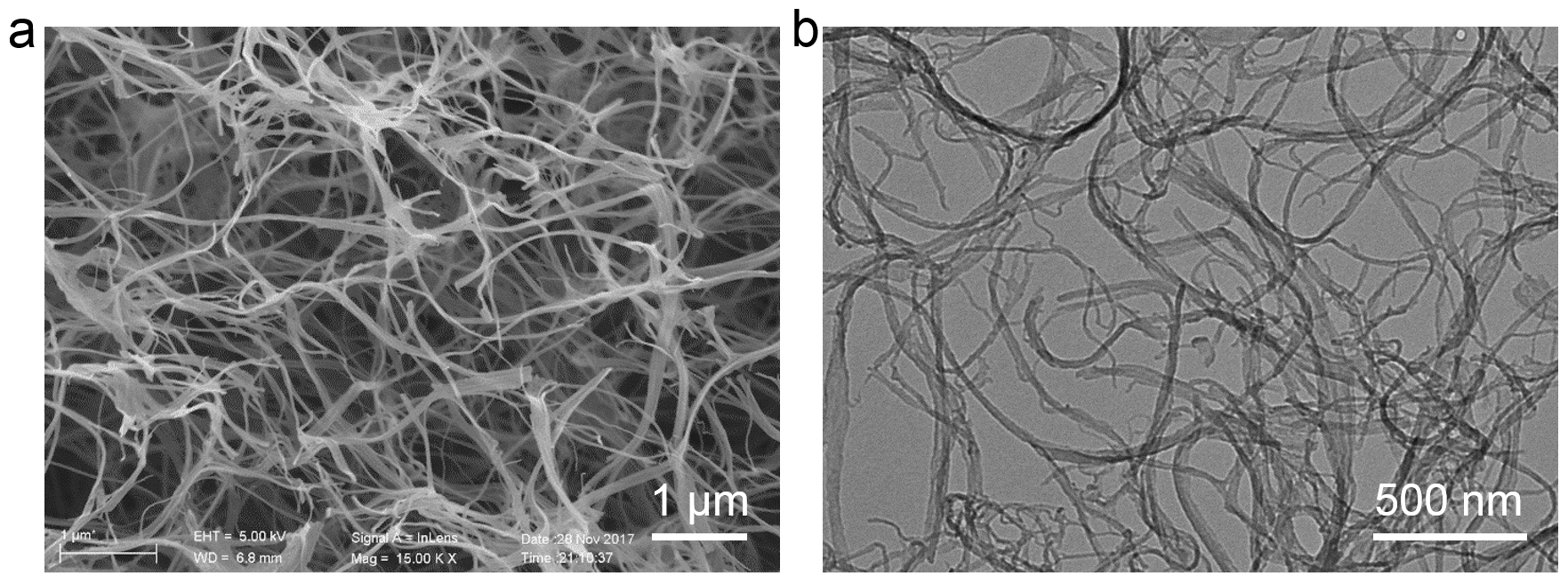


**Figure S1.** (a) SEM and (b) TEM images of BC-CNFs-400.


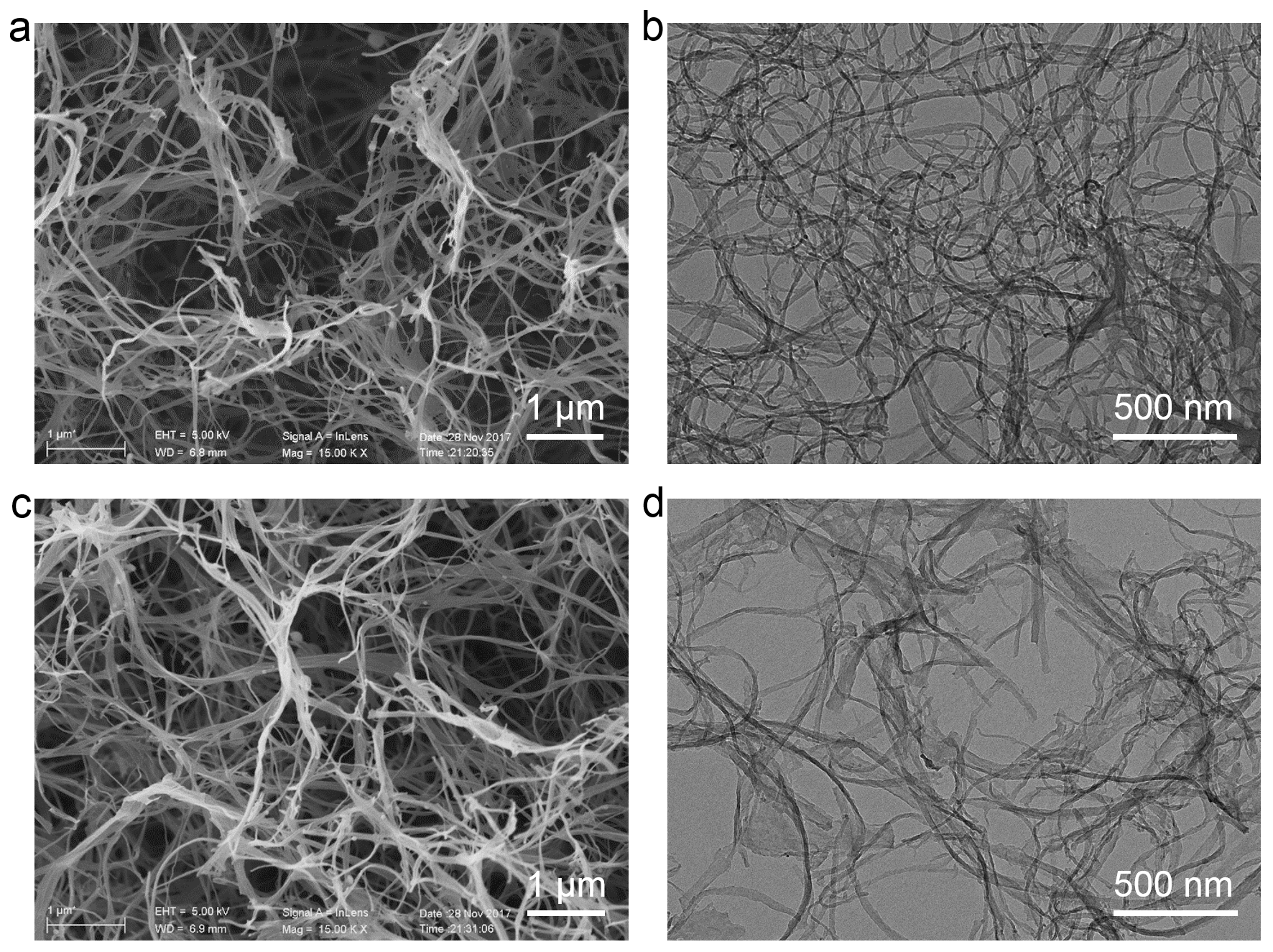


**Figure S2.** (a) SEM and (b) TEM images of BC-CNFs-600. (c) SEM and (d) TEM images of BC-CNFs-600-SO_3_H.


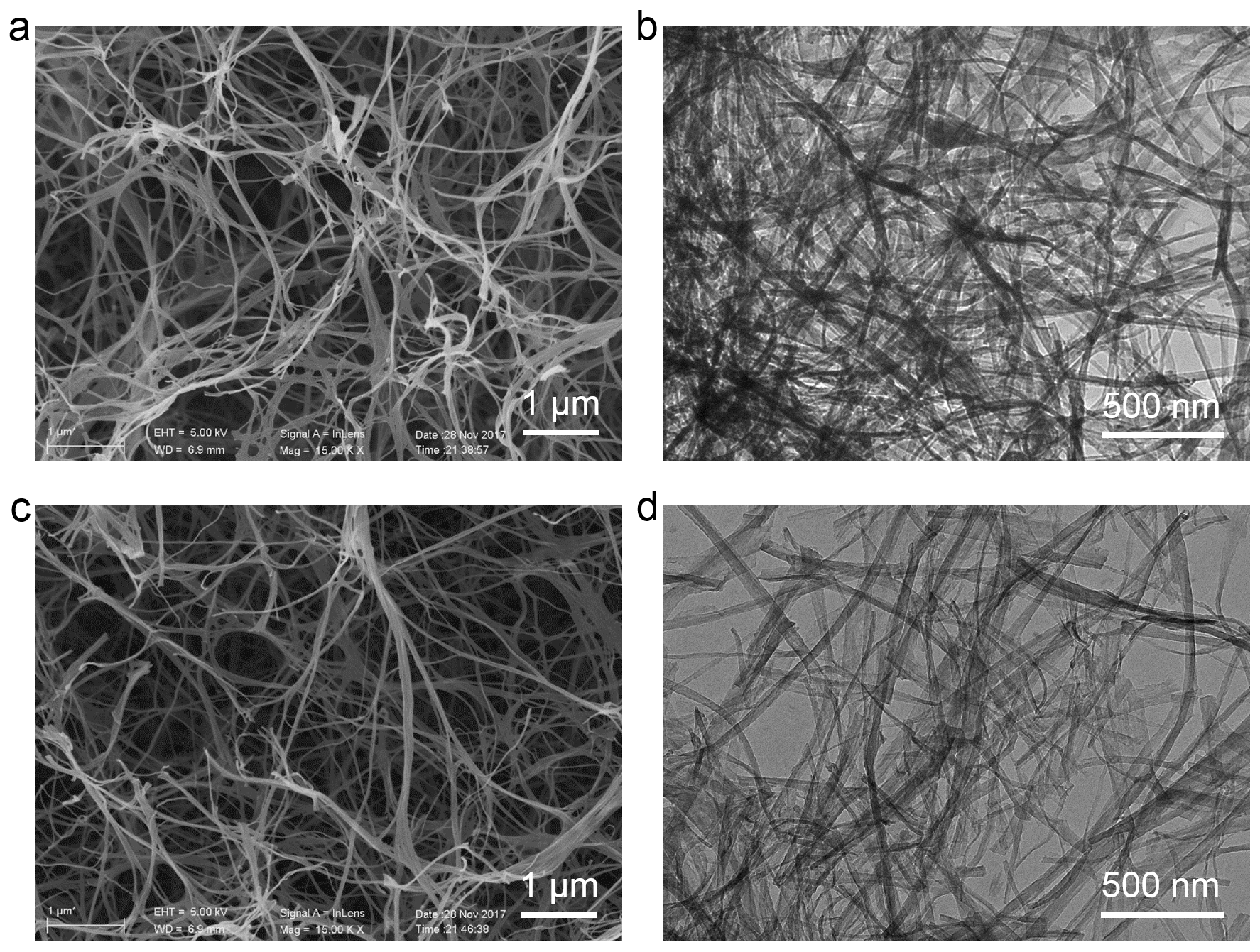


**Figure S3.** (a) SEM and (b) TEM images of BC-CNFs-800. (c) SEM and (d) TEM images of BC-CNFs-800-SO_3_H.


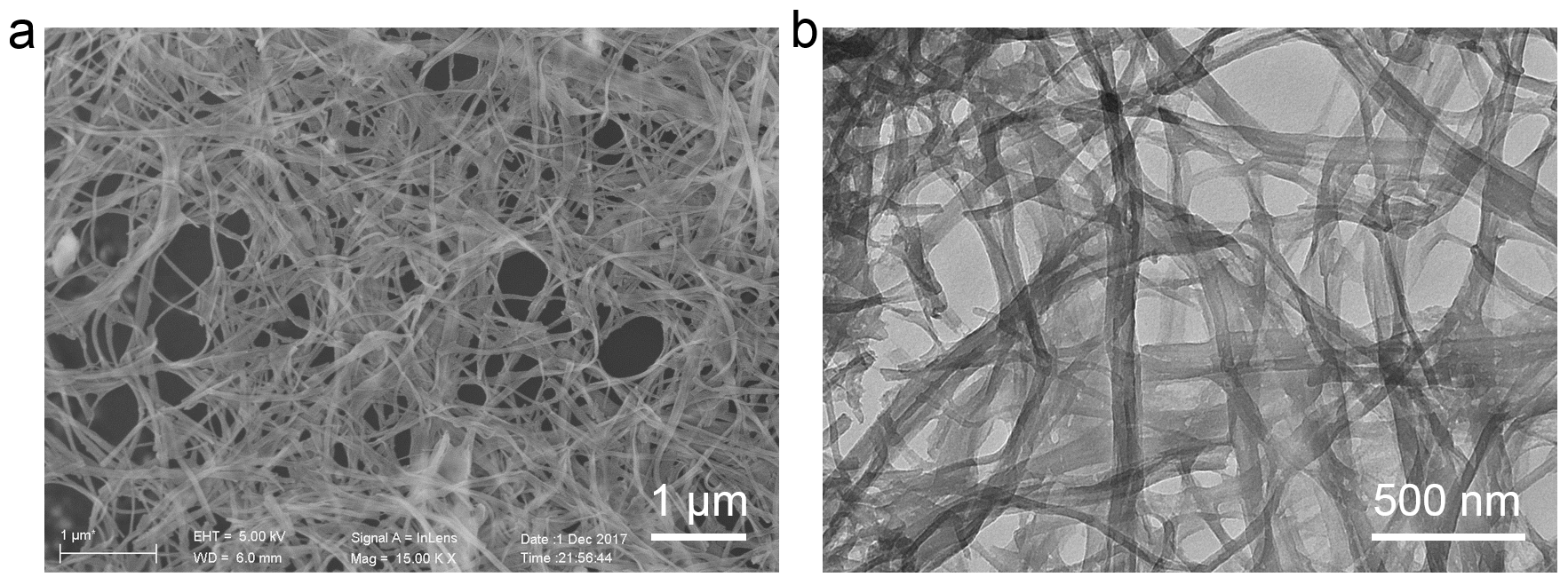


**Figure S4.** (a) SEM and (b) TEM images of BC-CNFs-400-SO_3_H*.


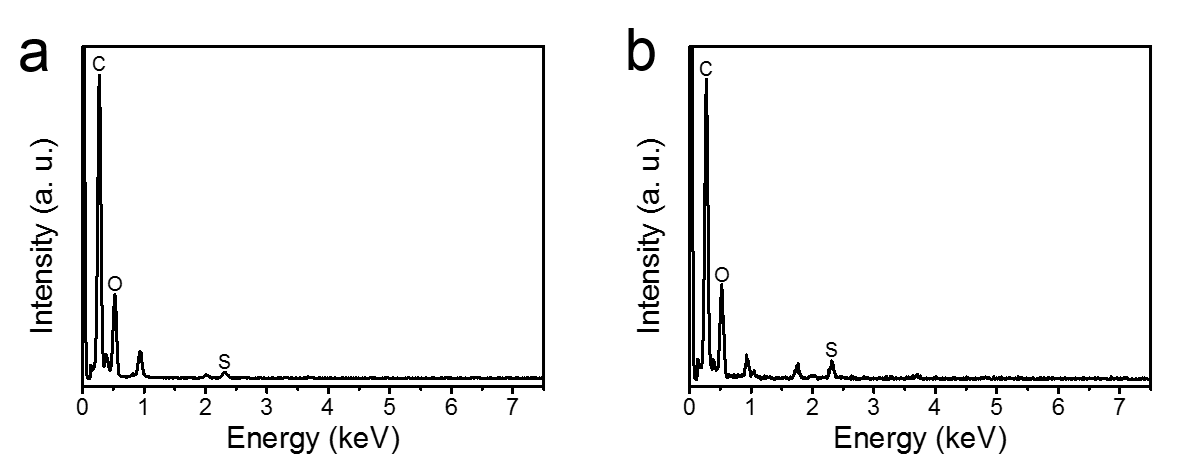


**Figure S5.** EDS spectra of (a) BC-CNFs-400-SO_3_H and (b) BC-CNFs-400-SO_3_H*.


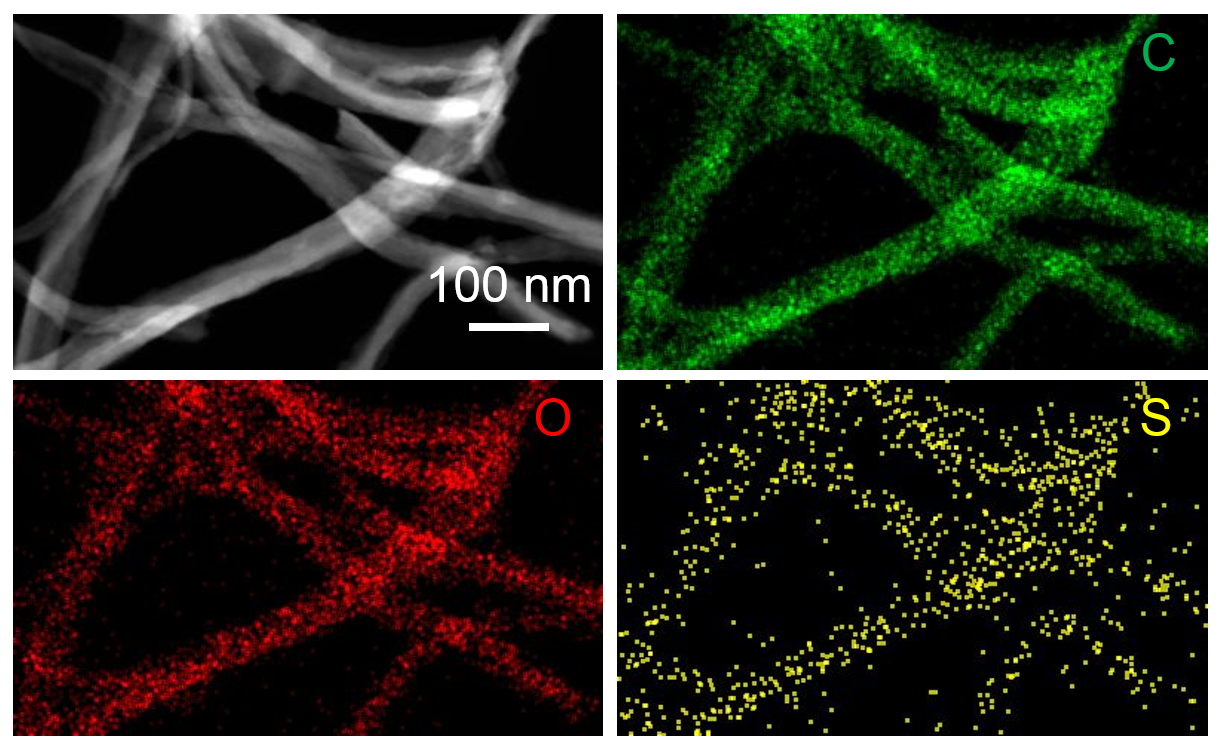


**Figure S6.** Elemental mapping images of BC-CNFs-400-SO_3_H network.


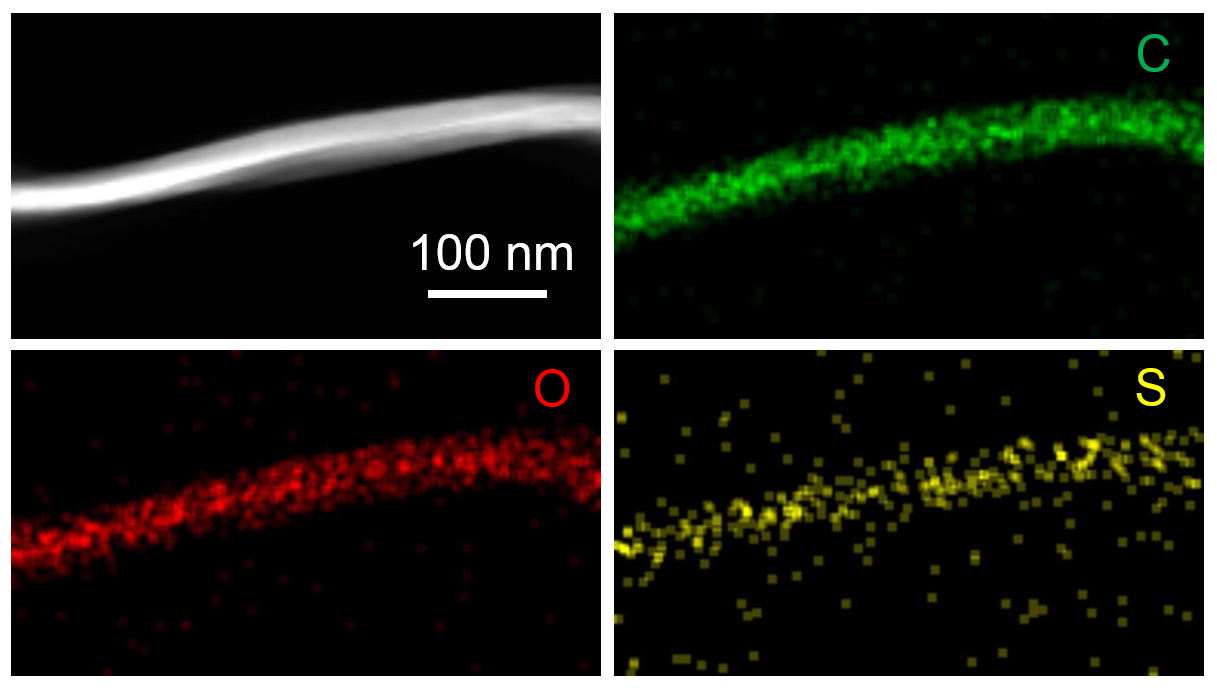


**Figure S7.** Elemental mapping images for an individual nanofiber of BC-CNFs-400-SO_3_H*.


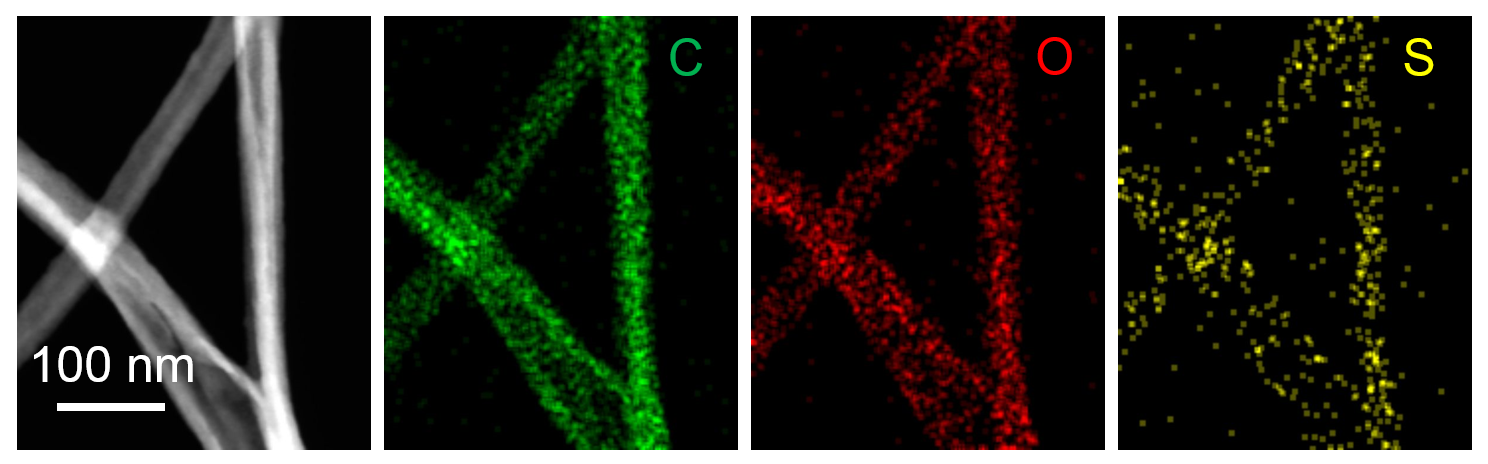


**Figure S8.** Elemental mapping of BC-CNFs-400-SO_3_H* network.


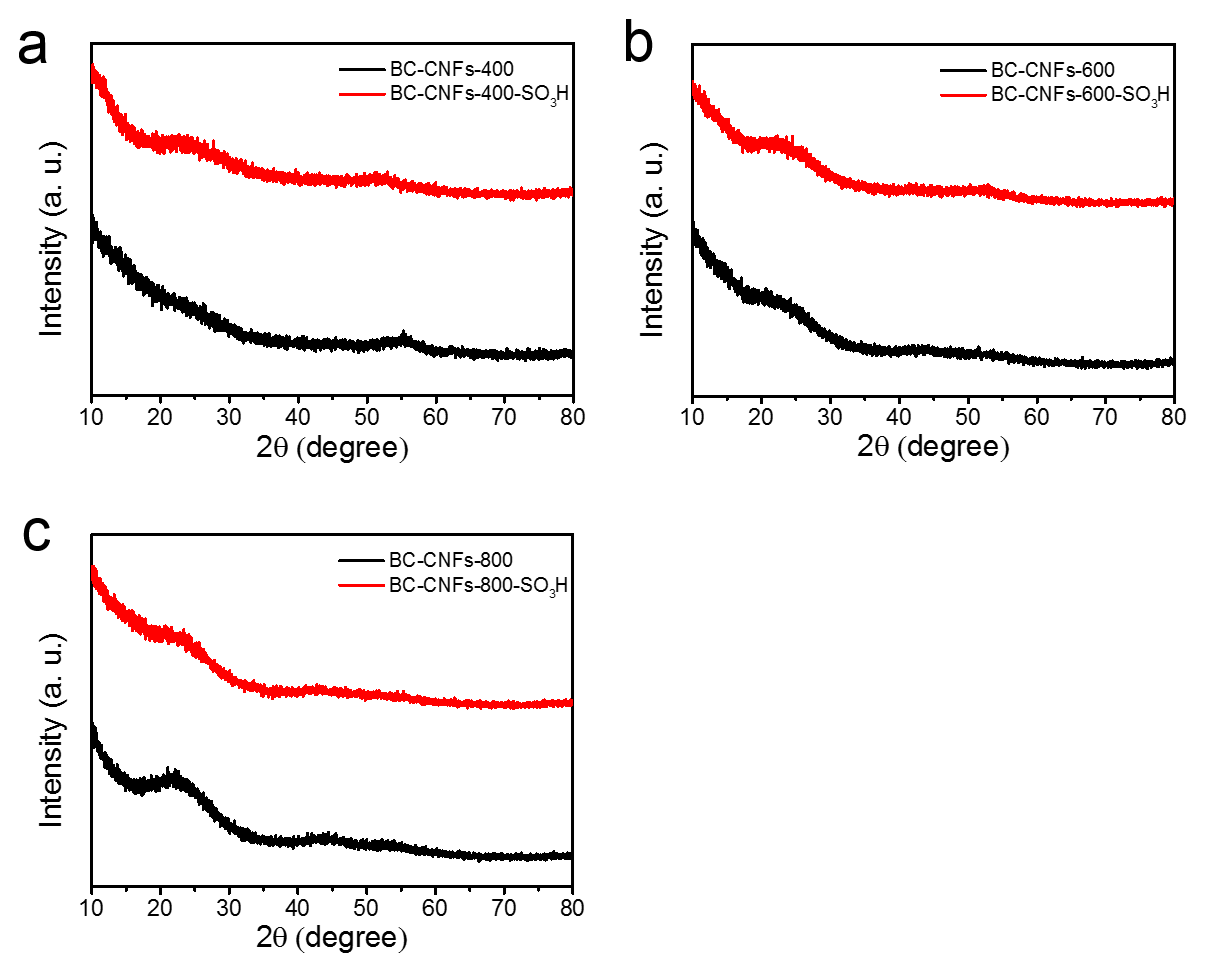


**Figure S9.** XRD patterns of (a) BC-CNFs-400 and BC-CNFs-400-SO_3_H, (b) BC-CNFs-600 and BC-CNFs-600-SO_3_H, and (c) BC-CNFs-800 and BC-CNFs-800-SO_3_H.


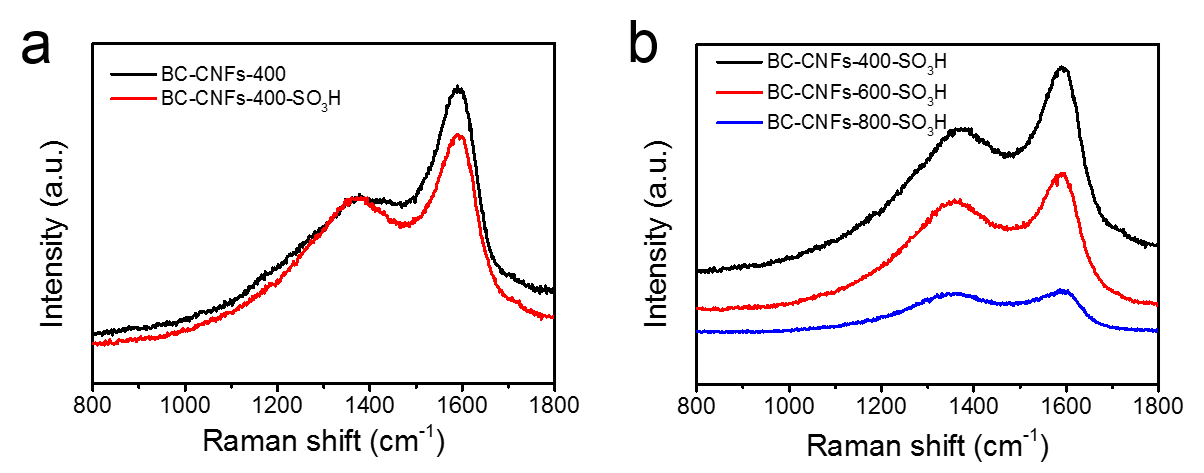


**Figure S10.** Raman spectra of (a) BC-CNFs-400 and BC-CNFs-400-SO_3_H, and (b) BC-CNFs-*x*-SO_3_H. The Raman spectra of the BC-CNFs-400 and BC-CNFs-*x*-SO_3_H exhibit two distinct signals assignable to the D (1350 cm^-1^, A_1g_ D breathing mode) and G bands (1580 cm^-1^, E_2g_ G mode), which is an indication of the presence of polycyclic aromatic carbon sheets in the carbon bulk [43, 44].


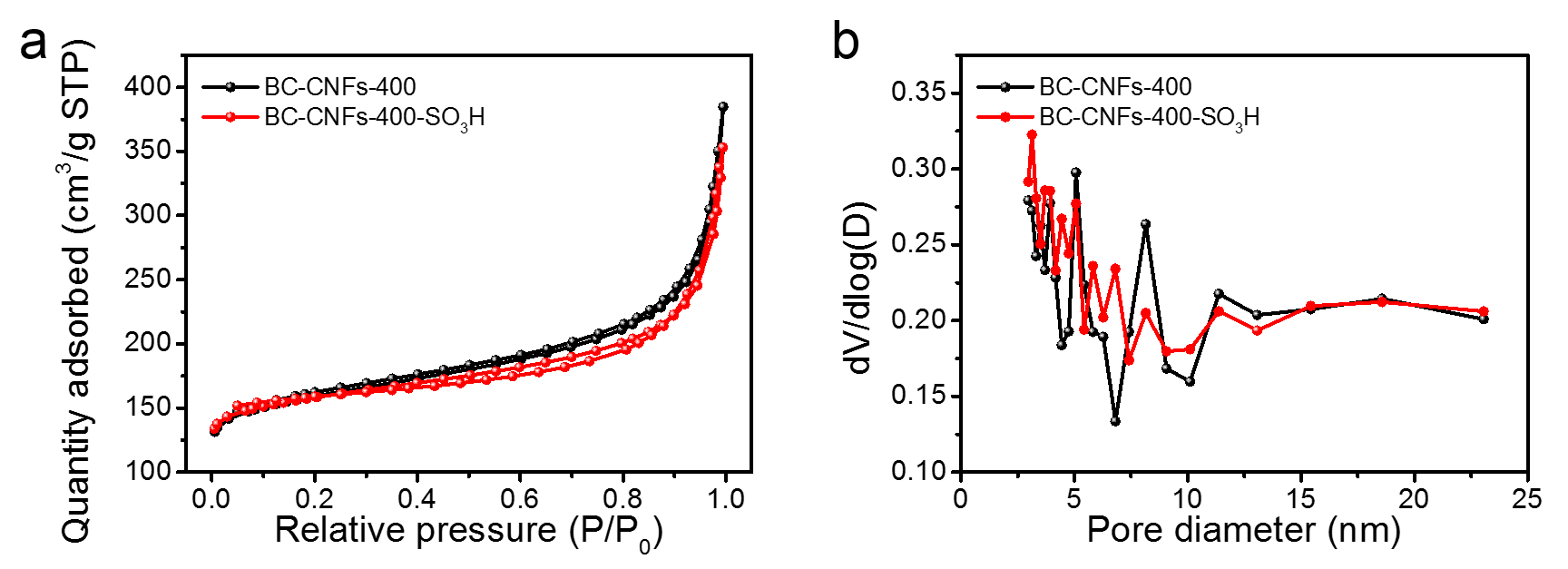


**Figure S11.** (a) N_2_ adsorption-desorption isotherms and (b) pore size distribution curves of BC-CNFs-400 and BC-CNFs-400-SO_3_H.


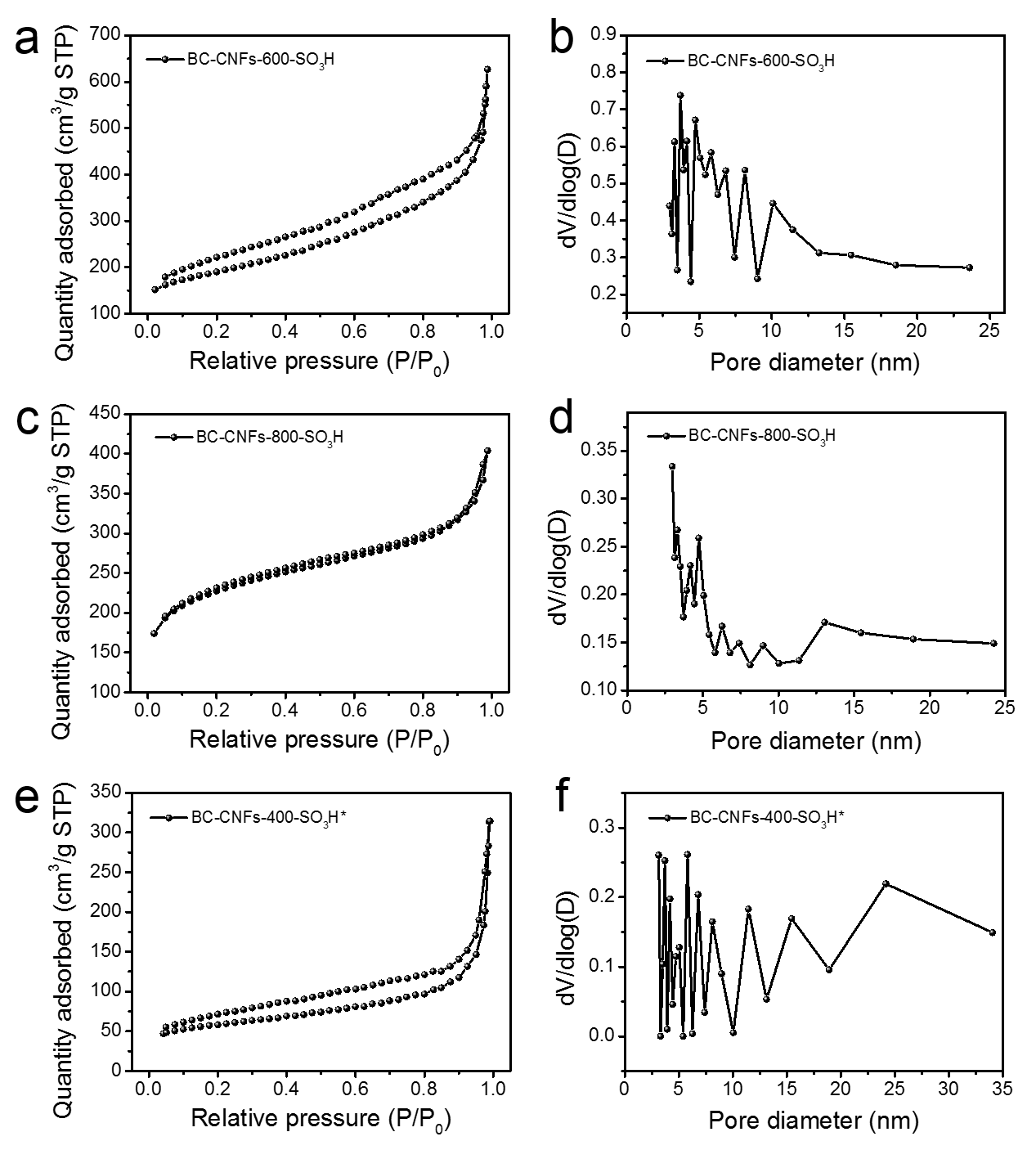


**Figure S12.** N_2_ adsorption-desorption isotherms of (a) BC-CNFs-600-SO_3_H, (c) BC-CNFs-800-SO_3_H and (e) BC-CNFs-400-SO_3_H*. Pore size distribution curves of (b) BC-CNFs-600-SO_3_H, (d) BC-CNFs-800-SO_3_H and (f) BC-CNFs-400-SO_3_H*.


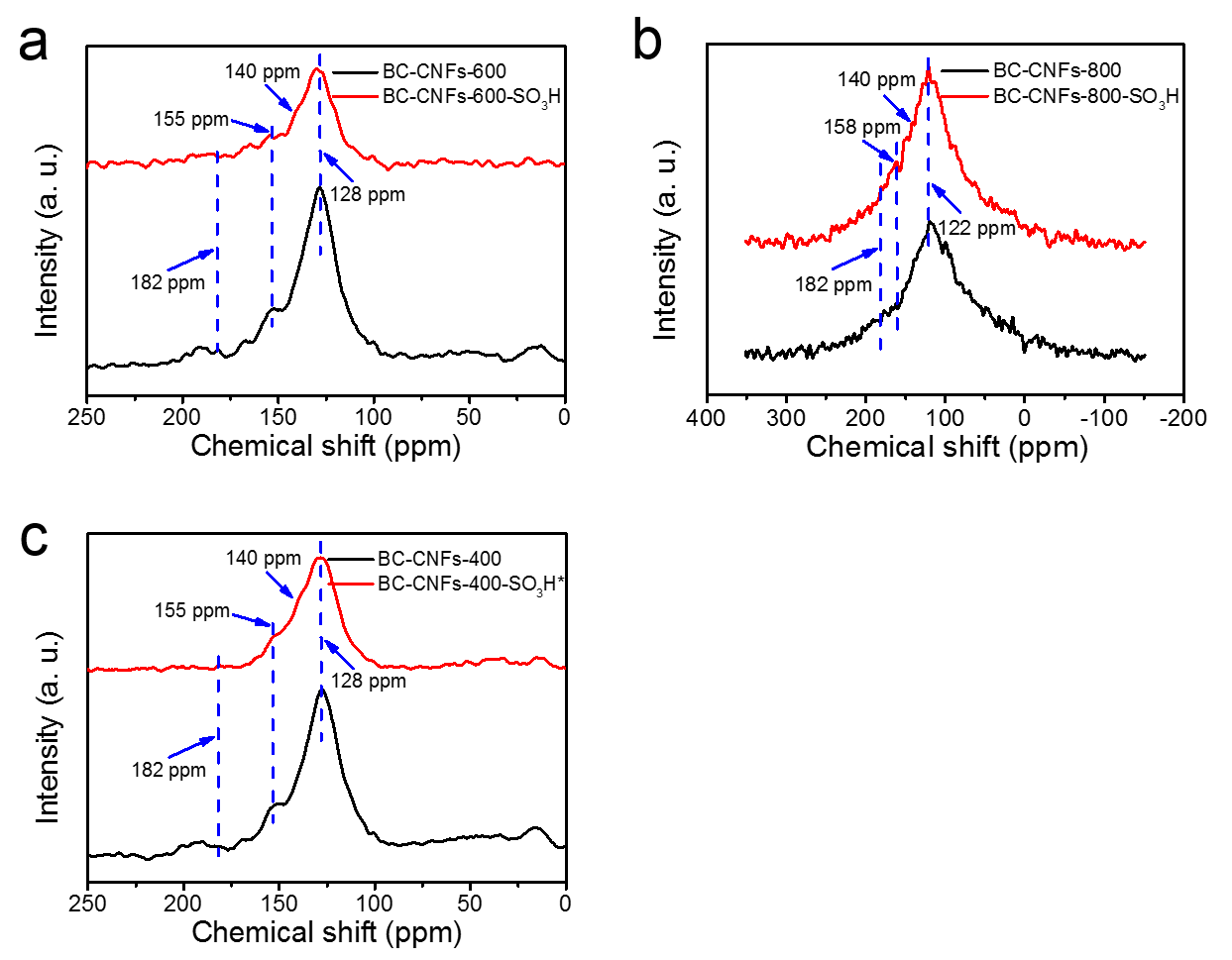


**Figure S13.** ^13^C MAS NMR spectra of (a) BC-CNFs-600 and BC-CNFs-600-SO_3_H, (b) BC-CNFs-800 and BC-CNFs-800-SO_3_H, and (c) BC-CNFs-400 and BC-CNFs-400-SO_3_H*.


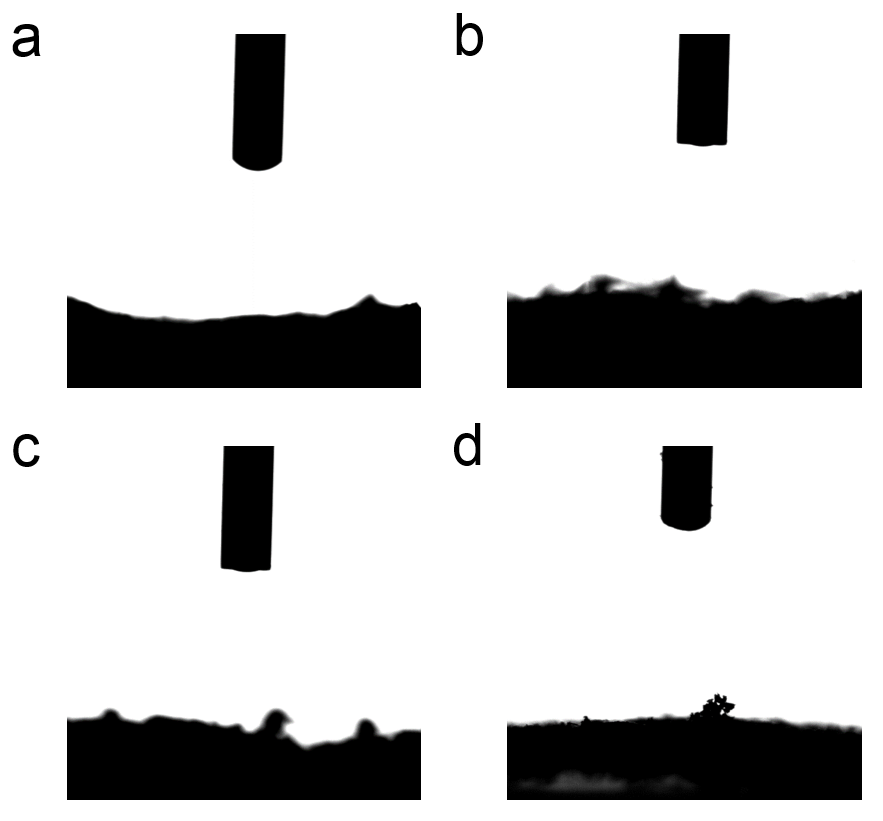


**Figure S14.** The measurement of contact angles with water for (a) BC-CNFs-400-SO_3_H, (b) BC-CNFs-600-SO_3_H, (c) BC-CNFs-800-SO_3_H, and (d) BC-CNFs-400-SO_3_H*.


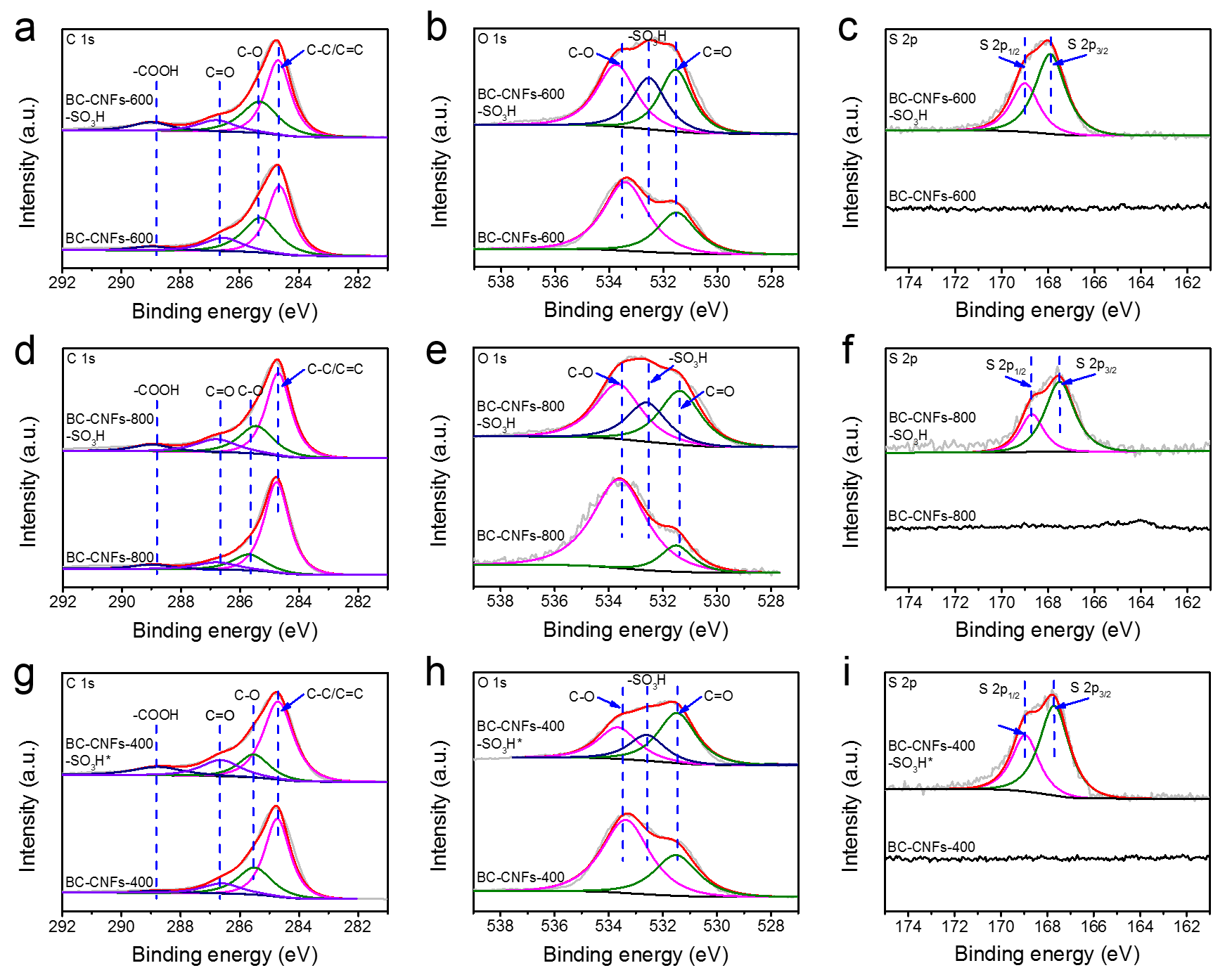


**Figure S15.** (a) C 1s, (b) O 1s and (c) S 2p high resolution XPS spectra of BC-CNFs-600 and BC-CNFs-600-SO_3_H. (d) C 1s, (e) O 1s and (f) S 2p high resolution XPS spectra of BC-CNFs-800 and BC-CNFs-800-SO_3_H. (g) C 1s, (h) O 1s and (i) S 2p high resolution XPS spectra of BC-CNFs-400 and BC-CNFs-400-SO_3_H*.


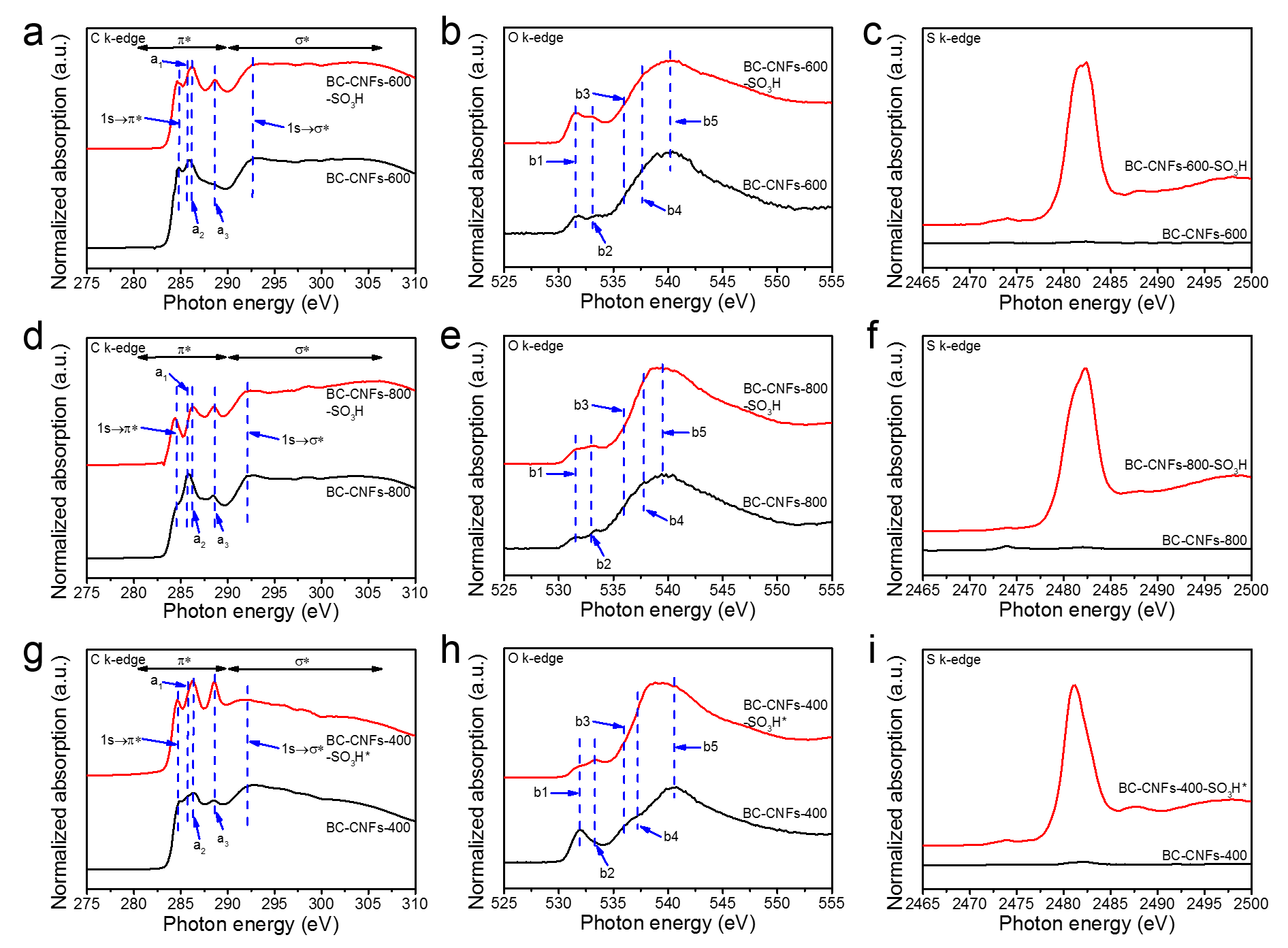


**Figure S16.** (a) C K-edge, (b) O K-edge and (c) S K-edge XAS spectra of BC-CNFs-600 and BC-CNFs-600-SO_3_H. (d) C K-edge, (e) O K-edge and (f) S K-edge XAS spectra of BC-CNFs-800 and BC-CNFs-800-SO_3_H. (g) C K-edge, (h) O K-edge and (i) S K-edge XAS spectra of BC-CNFs-400 and BC-CNFs-400-SO_3_H*.


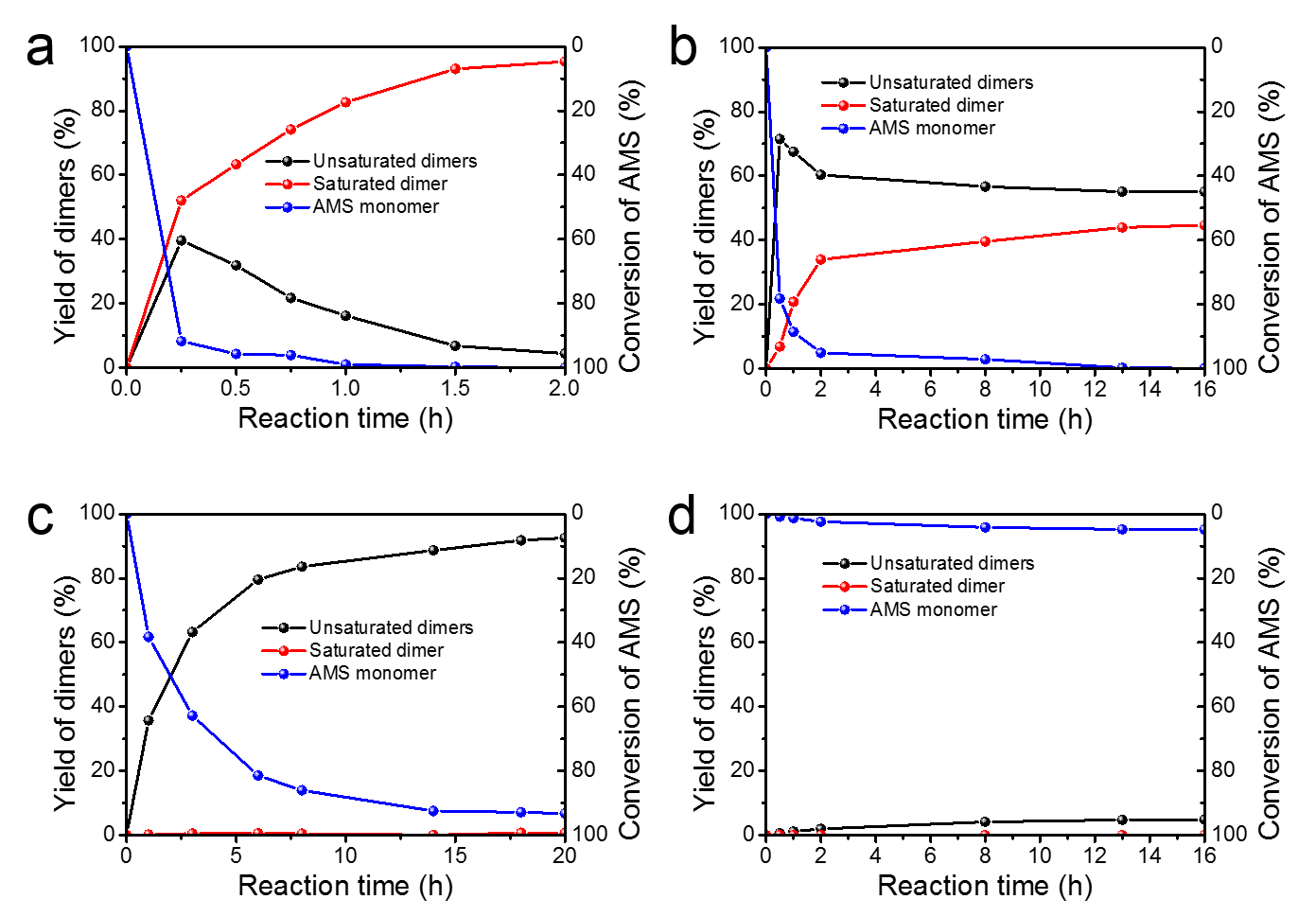


**Figure S17.** Time courses for AMS conversion and yields of unsaturated dimers and saturated dimers over (a) H_2_SO_4_, (b) Amberlyst-15, (c) BC-CNFs-600-SO_3_H and (d) Cellulose-600-SO_3_H.


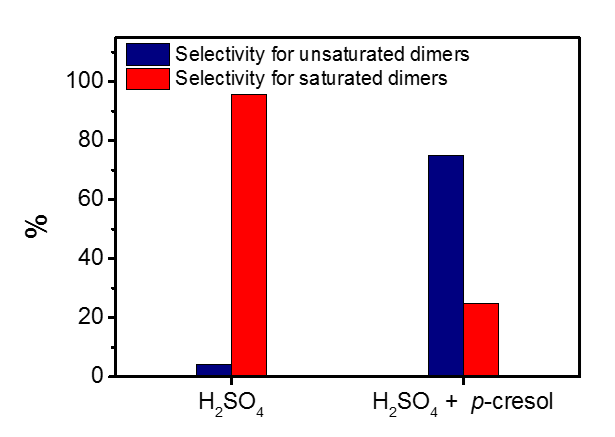


**Figure S18.** The dimerization of AMS over H_2_SO_4_ in the absence and presence of *p*-cresol (2.4 mL).


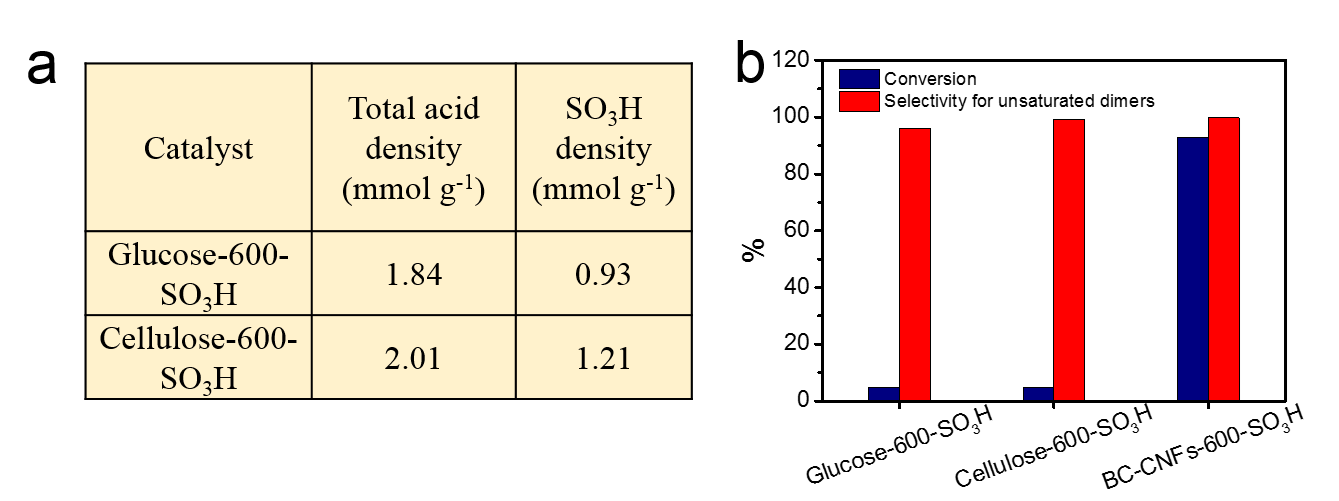


**Figure S19.** (a) Total acid density and SO_3_H density of glucose-600-SO_3_H and cellulose-600-SO_3_H. (b) AMS dimerization performance comparison among glucose-600-SO_3_H, cellulose-600-SO_3_H and BC-CNFs-600-SO_3_H.


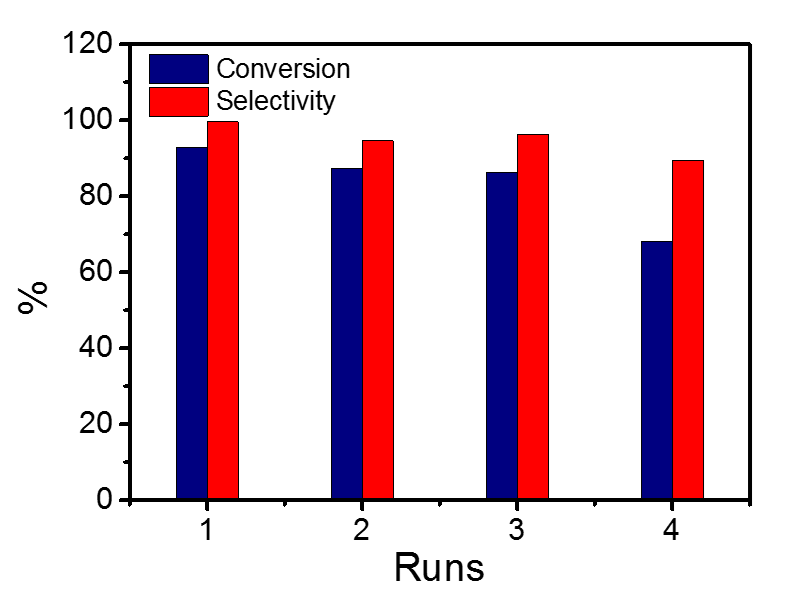


**Figure S20.** Recyclability performance of BC-CNFs-600-SO_3_H for AMS dimerization.


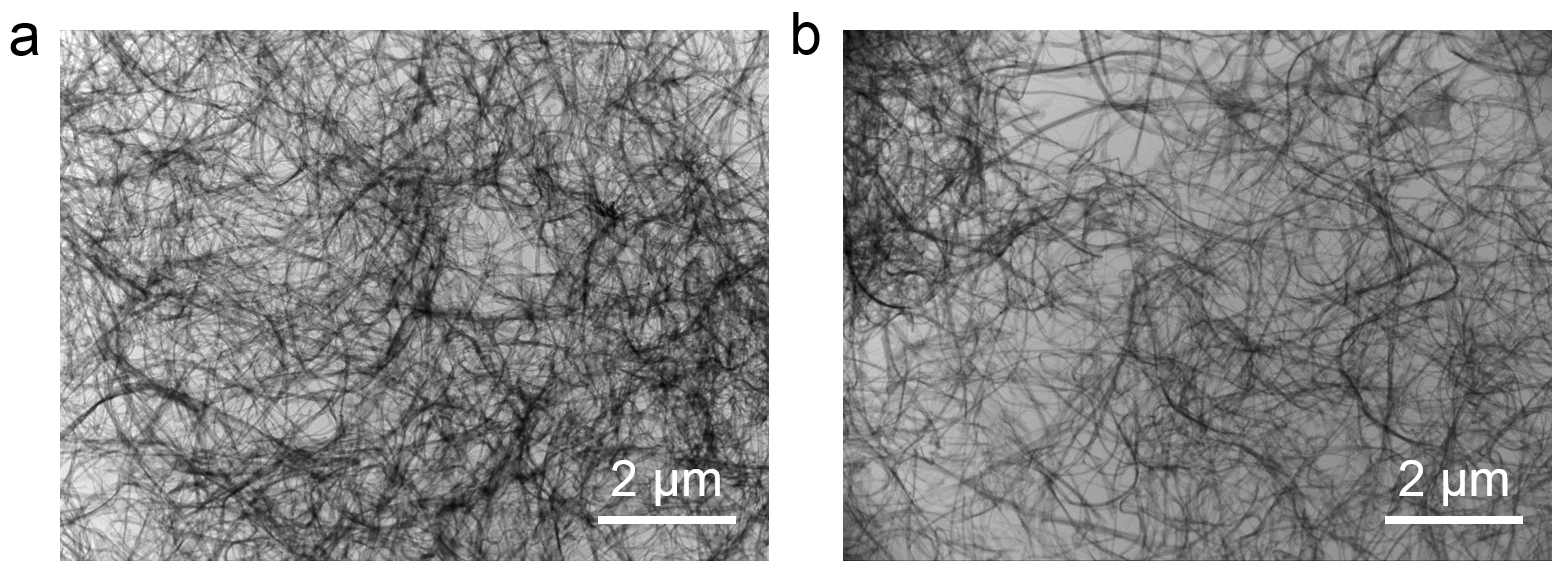


**Figure S21.** (a, b) TEM images of recycled BC-CNFs-600-SO_3_H after catalyzing AMS dimerization for four times.


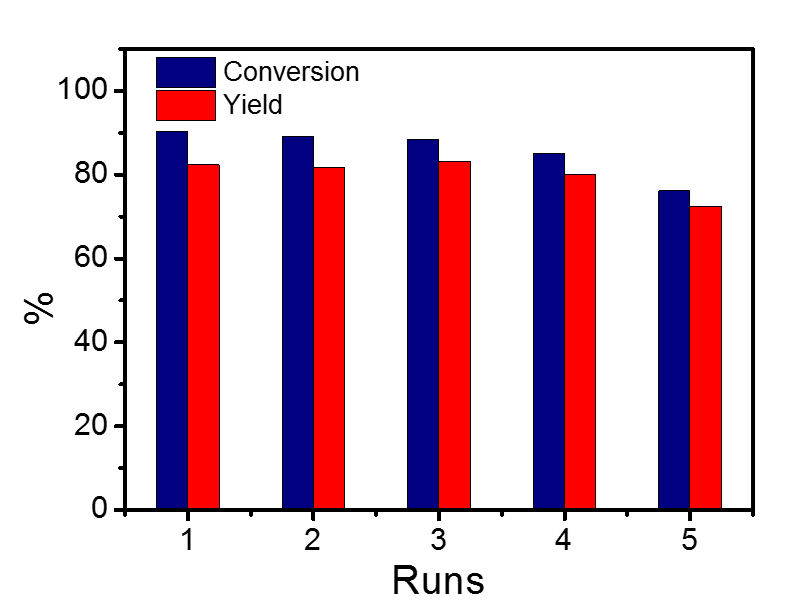


**Figure S22.** Recyclability performance of BC-CNFs-400-SO_3_H for esterification of oleic acid with methanol.

**
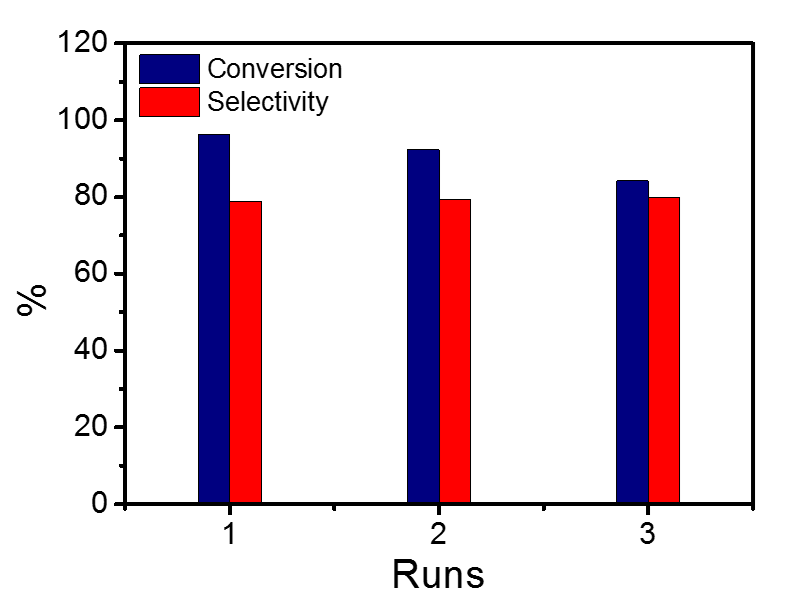
**

**Figure S23.** Recyclability performance of BC-CNFs-400-SO_3_H for pinacol rearrangement.


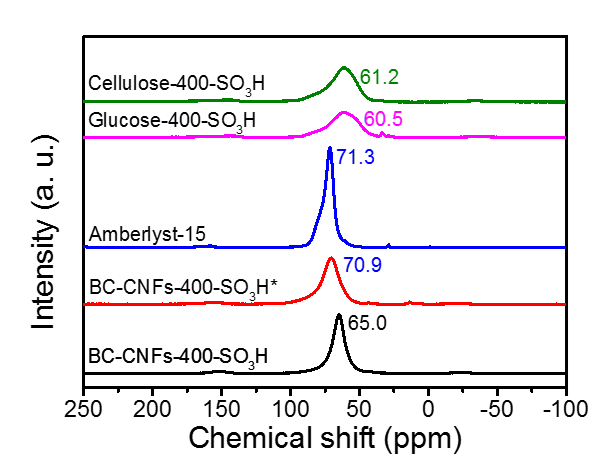


**Figure S24.** ^31^P MAS NMR spectra of BC-CNFs-400-SO_3_H* and some reference SACs.

The acid strength was monitored by reference to the ^31^P MAS NMR chemical shift of trimethylphosphine oxide (TMPO) chemically adsorbed on the SACs [9. 18]. In a typical measurement, 0.02 g of TMPO was dissolved in 10 ml of THF solution. Then, the resulting solution was mixed with 0.05 g of solid acids, which was subsequently subjected to a thorough dehydration treatment at 80 °C for 3 days. ^31^P MAS NMR spectra were recorded on a Bruker AVANCE 400WB NMR spectrometer (400 MHz).

The acid strength of BC-CNFs-400-SO_3_H* and some reference SACs was investigated using TEPO (triethylphosphine oxide) as a probe molecule in combination with ^31^P MAS NMR technique. The ^31^P chemical shift of TEPO chemisorbed on the acid site is sensitive to acid strength, and stronger acid strength will usually lead to larger ^31^P chemical shift of TEPO. The ^31^P MAS NMR spectra of Glucose-400-SO_3_H and Cellulose-400-SO_3_H exhibits signals at 60.5 and 61.2 ppm that can be assigned to TEPO adsorbed on SO_3_H acid sites. The signal at 65.0 ppm is observed in the ^31^P MAS NMR spectrum of BC-CNFs-400-SO_3_H. The downshift of the signal indicates that BC-CNFs-400-SO_3_H have stronger Brönsted acid strength than Glucose-400-SO_3_H and Cellulose-400-SO_3_H. In addition, BC-CNFs-400-SO_3_H* shows larger ^31^P chemical shift of 70.9 ppm, which is comparable to that of Amberlyst-15 (71.3 ppm) with high acid strength. The results indicates that BC-CNFs-400-SO_3_H* displays a high acid strength, very close to traditional strong SAC, i.e. Amberlyst-15 and much higher that reference Glucose-400-SO_3_H, Cellulose-400-SO_3_H and BC-CNFs-400-SO_3_H.


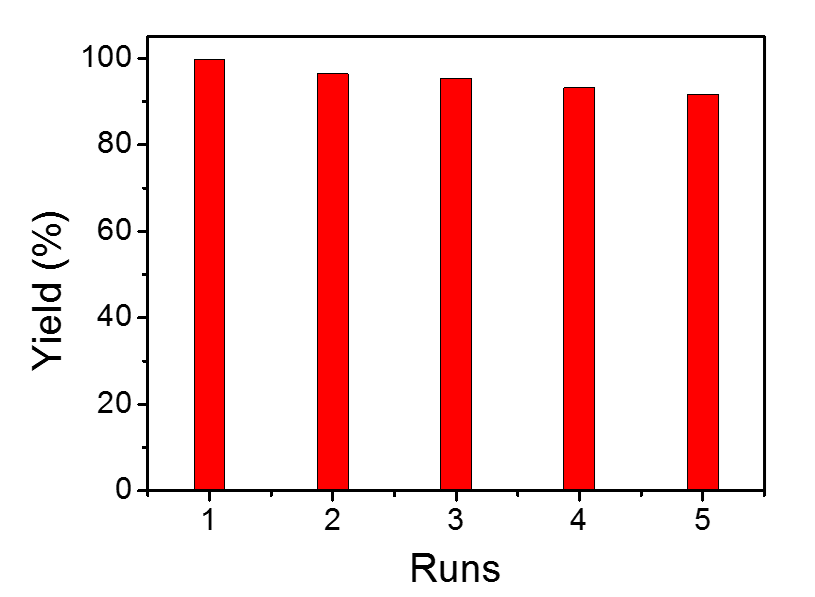


**Figure S25.** Recyclability performance of BC-CNFs-400-SO_3_H for hydrogenation of nitrobenzene.


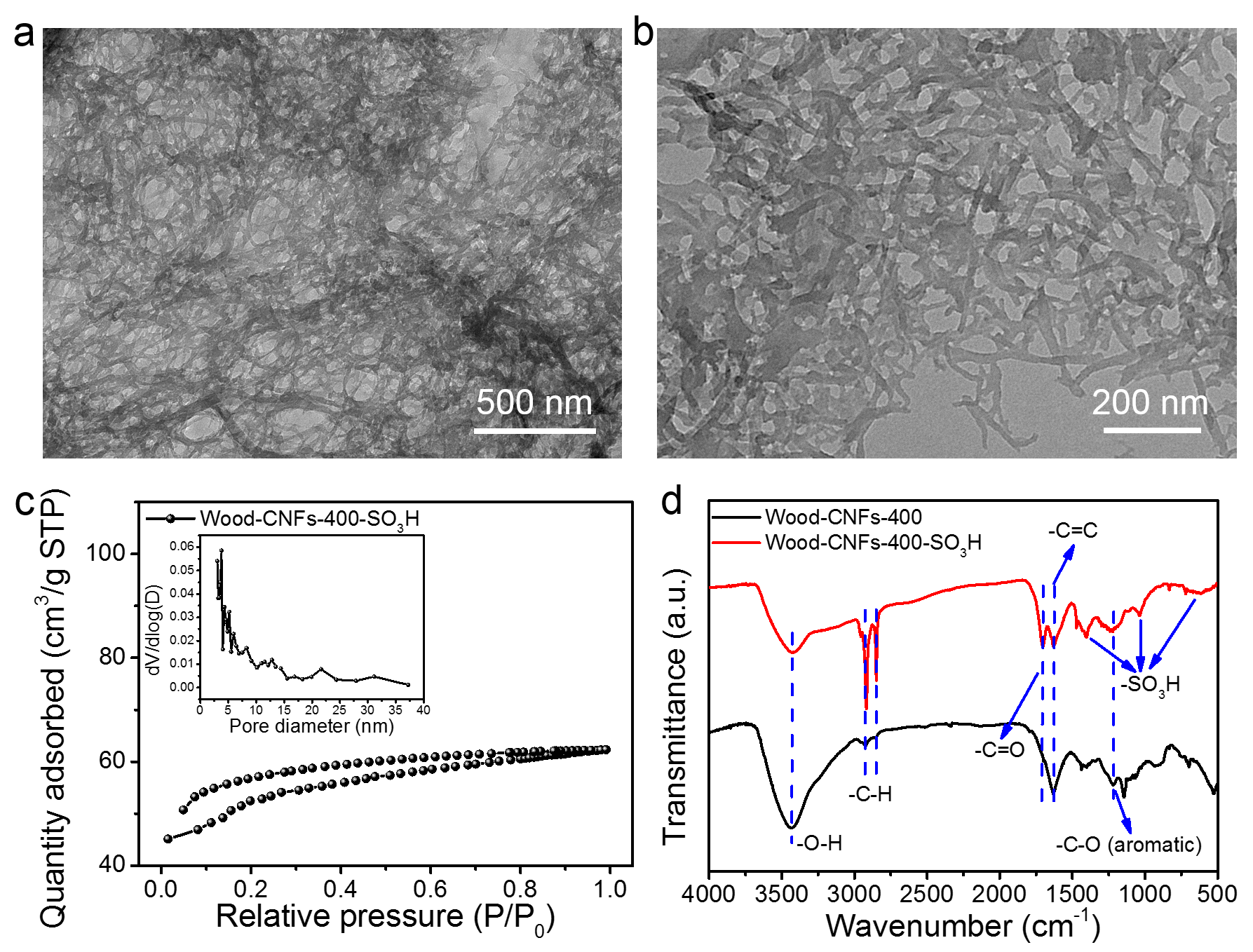


**Figure S26.** (a, b) TEM images of Wood-CNFs-400-SO_3_H. (c) N_2_ adsorption-desorption isotherms of Wood-CNFs-400-SO_3_H. Inset is corresponding pore size distribution curves of Wood-CNFs-400-SO_3_H. (d) FT-IR spectra of Wood-CNFs-400 and Wood-CNFs-400-SO_3_H.


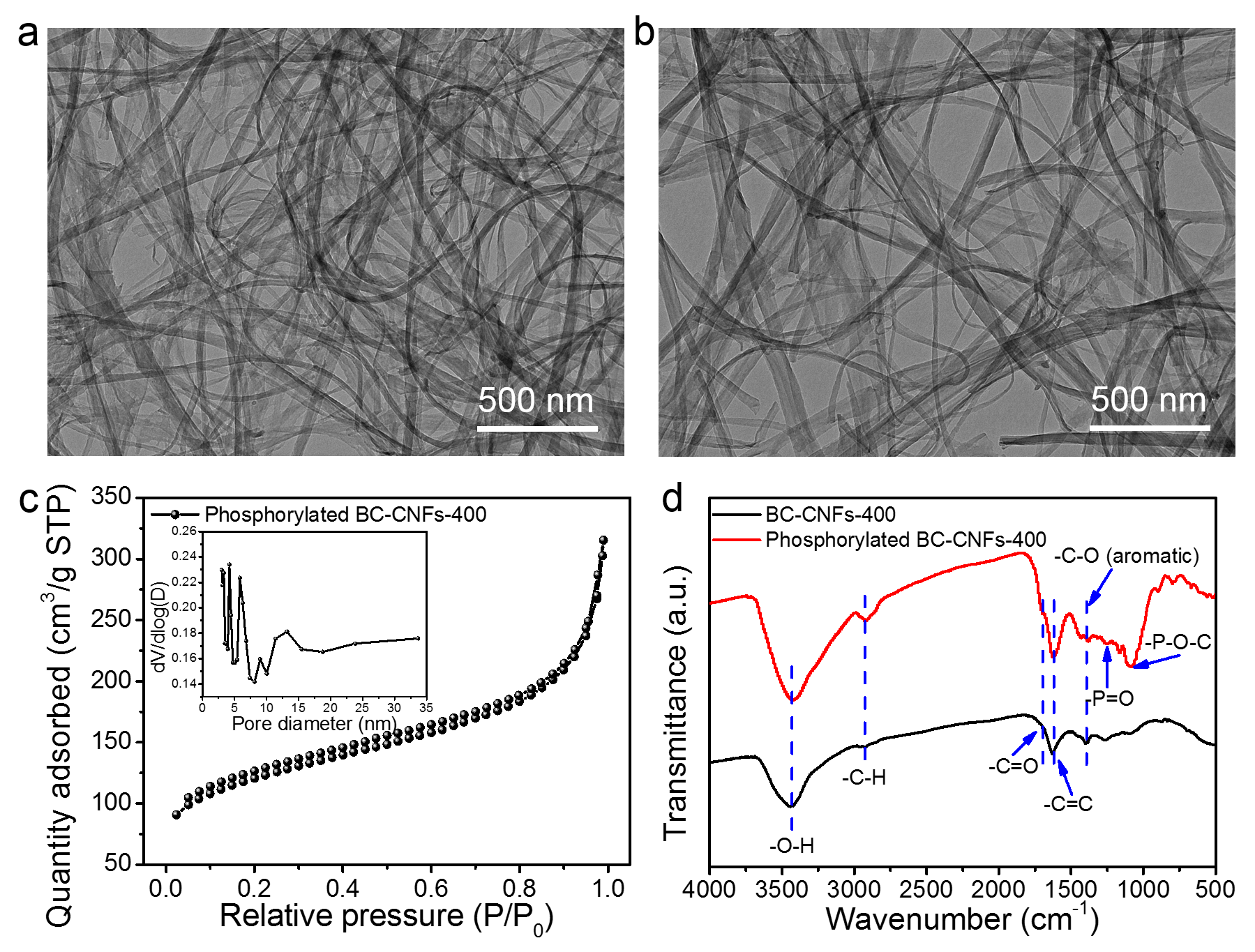


**Figure S27.** (a, b) TEM images of phosphorylated BC-CNFs-400. (c) N_2_ adsorption-desorption isotherms of phosphorylated BC-CNFs-400. Inset is corresponding pore size distribution curves of phosphorylated BC-CNFs-400. (d) FT-IR spectra of BC-CNFs-400 and phosphorylated BC-CNFs-400.

**Table S1.** Catalytic performance of the catalysts tested for synthesis of β‑Ketoenamines.

**
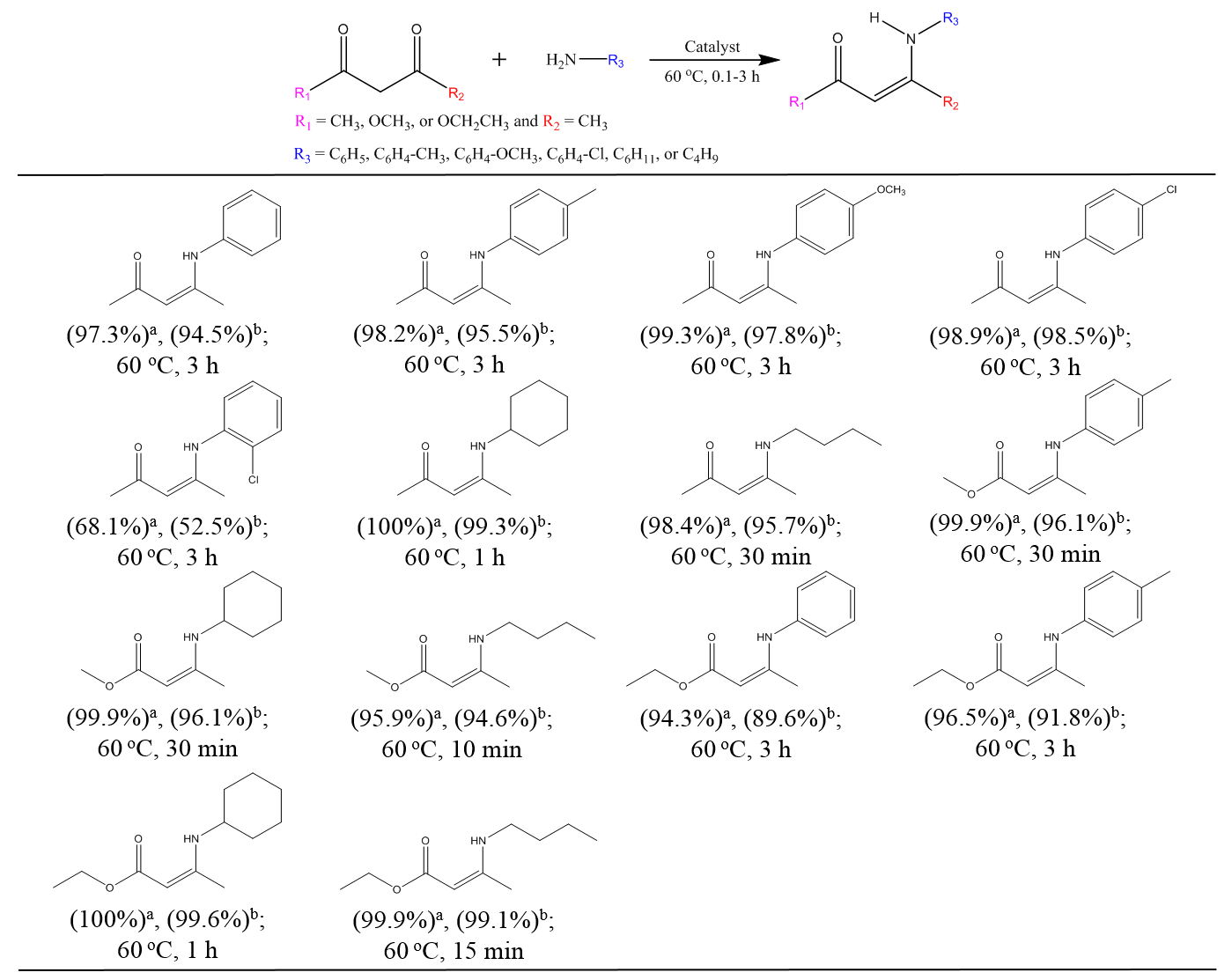
**

^a^ GC conversion; ^b^ GC yield.

**Table S2.** The comparison of catalytic performance of BC-CNF-400-SO_3_H and previously reported carbon-based catalysts for hydrogenation of nitrobenzene.

| Entry | Catalyst | Yield (%) | Reference |
| --- | --- | --- | --- |
| 1 | BC-CNFs-400-SO_3_H | 99.6^a^ | **This work** |
| 2 | BC-CNFs-400 | 78.6^a^ | **This work** |
| 3 | RGO | 94.2^b^ | *Chem. Commun.* **2011**, *47*, 2432-2434 |
| 4 | GO | 91.1^b^ | *Chem. Commun.* **2011**, *47*, 2432-2434 |
| 5 | N-doped mesoporous carbon-silica composite | 58.7^c^ | *Mater. Lett.* **2013**, *108*, 285-288 |
| 6 | N-doped mesoporous carbon | 88.6^c^ | *Mater. Lett.* **2013**, *108*, 285-288 |
| 7 | H_2_O_2_ treated activated carbon | 80.0^d^ | *J.Mol. Catal. A: Chem.* **2014**, *393*, 257-262 |
| 8 | Boron-doped onion-like carbon | 95.3^e^ | *Chem. Commun.* **2015**, *51*, 13086-13089 |
| 9 | Boron-doped CNTs | 98.3^e^ | *Chem. Commun.* **2015**, *51*, 13086-13089 |
| 10 | Nitric acid oxidized CNT | 91.2^f^ | *Phys. Chem. Chem. Phys.* **2015**, *17*, 1567-1571 |
| 11 | H_2_O_2_ functionalized CNT | 99.3^f^ | *Phys. Chem. Chem. Phys.* **2015**, *17*, 1567-1571 |
| 12 | Hydrothermal carbon | 57.9^g^ | *Angew. Chem. Int. Ed.* **2017**, *56*, 600-604 |

^a^5 mg catalyst, 0.3 g nitrobenzene, 1.5 equivalent hydrazine hydrate, 353 K, 4 h;

^b^10 mg catalyst, 0.5 g nitrobenzene, 2 mL hydrazine hydrate, reflux, 4 h;

^c^10 mg catalyst, 0.5 g nitrobenzene, 2 mL hydrazine hydrate, 373 K, 4 h;

^d^10 mg catalyst, 8.5 mmol nitrobenzene, 4 mL hydrazine hydrate, 373 K, 2 h;

^e^10 mg catalyst, 10 mmol (1.23 g) nitrobenzene, 4 equivalent hydrazine hydrate, 373 K,4 h;

^f^20 mg catalyst, 1.2 g nitrobenzene, 5.0 equivalent hydrazine monohydrate, 368 K,5 h;

^g^20 mg catalyst, 1.2 g nitrobenzene, 6.0 equivalent hydrazine monohydrate (3.4 g), 373 K, 4 h.
